# Quantification of nitrogen oxides emissions from build-up of pollution over Paris with TROPOMI

**DOI:** 10.1038/s41598-019-56428-5

**Published:** 2019-12-27

**Authors:** A. Lorente, K. F. Boersma, H. J. Eskes, J. P. Veefkind, J. H. G. M. van Geffen, M. B. de Zeeuw, H. A. C. Denier van der Gon, S. Beirle, M. C. Krol

**Affiliations:** 1 Wageningen University, Environmental Sciences Group, Wageningen, The Netherlands; 2 Royal Netherlands Meteorological Institute, R&D Satellite Observations, De Bilt, The Netherlands; 3 Delft University of Technology, Delft, The Netherlands; 4 TNO, Department of Climate, Air and Sustainability, Utrecht, The Netherlands; 5 Max-Planck-Institut für Chemie, Mainz, Germany

**Keywords:** Atmospheric chemistry, Environmental monitoring

## Abstract

Nitrogen dioxide (NO_2_) is a regulated air pollutant that is of particular concern in many cities, where concentrations are high. Emissions of nitrogen oxides to the atmosphere lead to the formation of ozone and particulate matter, with adverse impacts on human health and ecosystems. The effects of emissions are often assessed through modeling based on inventories relying on indirect information that is often outdated or incomplete. Here we show that NO_2_ measurements from the new, high-resolution TROPOMI satellite sensor can directly determine the strength and distribution of emissions from Paris. From the observed build-up of NO_2_ pollution, we find highest emissions on cold weekdays in February 2018, and lowest emissions on warm weekend days in spring 2018. The new measurements provide information on the spatio-temporal distribution of emissions within a large city, and suggest that Paris emissions in 2018 are only 5–15% below inventory estimates for 2011–2012, reflecting the difficulty of meeting NO_x_ emission reduction targets.

## Introduction

Nitrogen oxides (NO_x_ = NO + NO_2_), mostly a product of combustion processes, play a key role in tropospheric chemistry, and influence air quality and atmospheric radiative forcing^1^. Nitrogen oxides are short-lived (NO_x_ lifetime of 1–12 hours)^2^, but their photochemical processing leads to longer lasting effects via the formation of ozone^3^ and aerosols, as well as acid rain^4^. In response, European Union legislation establishes a maximum acceptable nitrogen dioxide (NO_2_) concentration in ambient air of 40 μg/m^3^. In 2016, this annual limit for NO_2_ was widely exceeded across Europe^5^. For example, 1.4 million Parisians were exposed to NO_2_ levels exceeding the limit, mostly because of strong emissions from road traffic, and from residential and commercial combustion^6^, despite sizeable reductions in emissions reported over the last decade^7^. Reliable and comprehensive emission estimates are needed to evaluate air quality mitigation strategies and as input to models simulating and forecasting air pollution. Satellite measurements provide a comprehensive perspective on the spatial distribution^8,9^ and temporal evolution^10,11^ of global emissions. Such emission estimates are still limited in their spatial and temporal resolution. There remains a clear need for accurate emission estimates at the sub-urban scale on a day-to-day basis.

Here we report on the first NO_x_ emission estimates from new NO_2_ column measurements by the recently launched TROPOMI^12^ instrument on the Sentinel-5 Precursor (S5P) mission. TROPOMI extends the data records obtained from SCIAMACHY (2002–2012), GOME-2 (since 2007), and OMI (since 2004), and is the preparatory mission for Sentinel-5, due for launch in the 2020 s. TROPOMI is a spectrometer measuring direct and reflected sunlight at around 13:30 hrs local time in ultraviolet and visible bands, as well as radiances and irradiances in the near- and shortwave infrared. Besides NO_2_, these spectral bands allow the observation of ozone, carbon monoxide^13^, sulfur dioxide, formaldehyde, and methane, as well as aerosol and cloud properties. Satellite data quality has gradually increased over the last decade, but the very high spatial resolution of 3.5 × 7 km^2^, (across × along track) and improved signal-to-noise offered by TROPOMI^14^ is a major step forward. The spatial resolution of TROPOMI is more than 10 × better than its predecessor (the Ozone Monitoring Instrument – OMI^15^). This greatly improves the potential to detect pollution in broken cloud fields, to pinpoint small-scale emission sources, and estimate very localized emissions from industry or fires.

In this study we developed a method to estimate the NO_x_ emissions directly from the build-up of TROPOMI NO_2_ columns^14,16^ observed over Paris on single clear-sky days, without the need for complex inversions with a chemistry-transport model^17^. Our method allows inferring the NO_x_ emissions from the observed NO_2_ in air advected over the city, provided that wind speed and wind direction are known with good accuracy. Under non-stagnant conditions, the chemical decay of NO_2_ in the boundary layer is of minor importance given the short time it takes for an air parcel to cross the Paris Metropolitan area relative to the chemical lifetime^2,7,17^. The quality of the NO_2_ retrievals from TROPOMI is such, that it is not necessary to reduce noise by averaging satellite NO_2_ distributions for a particular wind direction sector first^14,17^. This avoids errors associated with interpreting average patterns based on an ensemble of individual plumes from different days with different wind directions and wind speeds. Instead, we directly analyse the build-up of NO_2_ pollution over the city from an individual TROPOMI orbit, thereby achieving one or sometimes even two estimates of city-wide NO_x_ emissions on a particular day.

**Figure 1:**
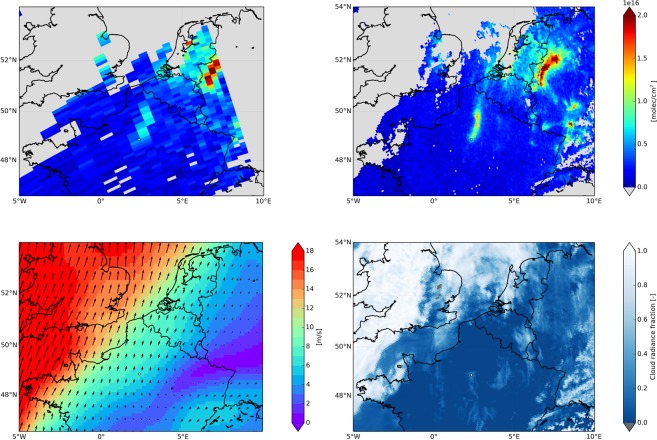
Tropospheric NO_2_ vertical column on 17 April 2018 from (**a**) OMI (DOMINO v2), and (**b**) TROPOMI. (**c**) ECMWF Era Interim 10 meter wind speed on 17 April 2018 at 12:00 UTC, (**d**) TROPOMI cloud radiance fraction. In (**a**,**b**) data has been filtered for cloud radiance fraction (lower than 0.5) and surface albedo (lower than 0.3), and grey areas indicate cloudiness or no data (in the case of OMI). Measurement time was approximately 12:15 UTC for both OMI and TROPOMI. Paris is indicated as a small white circle in the panels, with the Paris city centre at 48.86°N, 2.36°E.

Figure 1 illustrates that the TROPOMI measurements present an improvement over OMI, with TROPOMI clearly capturing the details of a NO_2_ pollution plume originating from Paris and blown to the north on 17 April 2018. Indeed, Fig. 1(c) shows that wind direction and speed from the European Centre for Medium Range Weather Forecasts^18^ indicate a southerly flow on this day, which was mostly cloud-free at the overpass (Fig. 1(d)). The tropospheric NO_2_ columns from TROPOMI are per definition representative for the vertically integrated NO_2_ concentrations between the surface and the tropopause, and they are directly linked to the NO_x_ emissions. We select measurements taken under mostly cloud-free conditions, when TROPOMI is having good sensitivity to enhanced NO_2_ concentrations in the polluted boundary layer.

## Results

### Validation of TROPOMI NO_2_ over Paris

We validated the TROPOMI NO_2_ columns over Paris. We compared the TROPOMI columns against a set of coincident NO_2_ columns inferred from *in situ* (AirParif^19^) NO_2_ measurements taken on the Eiffel Tower using information on boundary layer height^20^. The hourly NO_2_ concentration values closest in time to the TROPOMI overpass were converted into surface concentrations *C*_*g*_ representative for the Eiffel Tower pixel (by multiplying with 1.4, the mean ratio between NO_2_ surface and Eiffel Tower concentrations Fig. 1(b) in Dieudonné *et al*.^20^), effectively accounting for the vertical gradient of NO_2_ in the boundary layer. We then applied the empirical relationship between surface and column NO_2_ values established from 2 years of coincident column and *in situ* measurements over Paris. This empirical relationship relates NO_2_ surface concentrations to the NO_2_ column (*N*_*AP*_) via the boundary layer height^20^:

1 $${N}_{AP}=K(0.244\,h({C}_{g}-1.38)+0.184\,({C}_{g}-2.83))$$

with *K* a constant factor that converts 1 μg/m^3^ in a 1 km deep boundary layer into a column of 1.31 × 10^15^ molec.cm^−2^, *C*_*g*_ the surface NO_2_ concentration (in μg/m^3^), and *h* the boundary layer height in km (from ECMWF). The scaling factors in Eq. (1) have been determined by fitting the tropospheric NO_2_ columns against NO_2_ surface concentration for different boundary layer height classes, and show that the NO_2_ columns scale progressively with increasing boundary layer height^20^.

**Figure 2:**
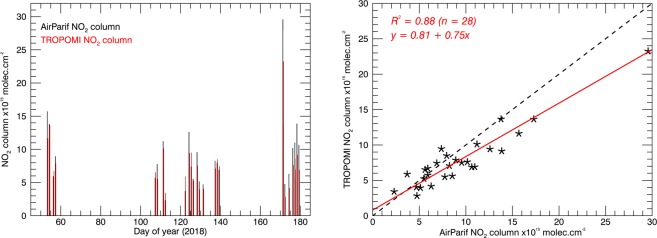
Comparison of tropospheric NO_2_ columns from AirParif (calculated with Eq. (1)) and TROPOMI over the Eiffel Tower. The left panel shows a time series of the clear-sky columns between February and July 2018. The right panel shows a scatter plot and the results of a reduced major axis regression analysis of TROPOMI vs. AirParif. The average distance of the TROPOMI pixel centres to the Eiffel Tower was 2.6 km.

By applying the above procedure, we obtained 28 ‘AirParif’ NO_2_ columns measured within 30 minutes of the TROPOMI observations over the Eiffel Tower. On one day, 24 April 2018, there were no NO_2_ measurements available from AirParif. The comparison shown in Fig. 2 suggests excellent agreement (R^2^ = 0.88) between the TROPOMI and AirParif columns. A reduced major axis regression suggests that TROPOMI has a small, systematic offset of +0.8 10^15^ molec. cm^−2^ and a slope of 0.75 relative to the AirParif columns. On average TROPOMI NO_2_ columns are lower than those from AirParif by 10–15%. The multiplicative component of the bias (the slope of 0.75) indicates that the increases in NO_2_ columns over Paris are underestimated by the same margin. We correct for the multiplicative component of the bias, most likely caused by air mass factor errors, by scaling up the observed NO_2_ columns with a factor of 1.33. A similar low bias was reported by Griffin *et al*.^21^.

#### NO_2_ build-up in air advected over Paris

**Figure 3:**
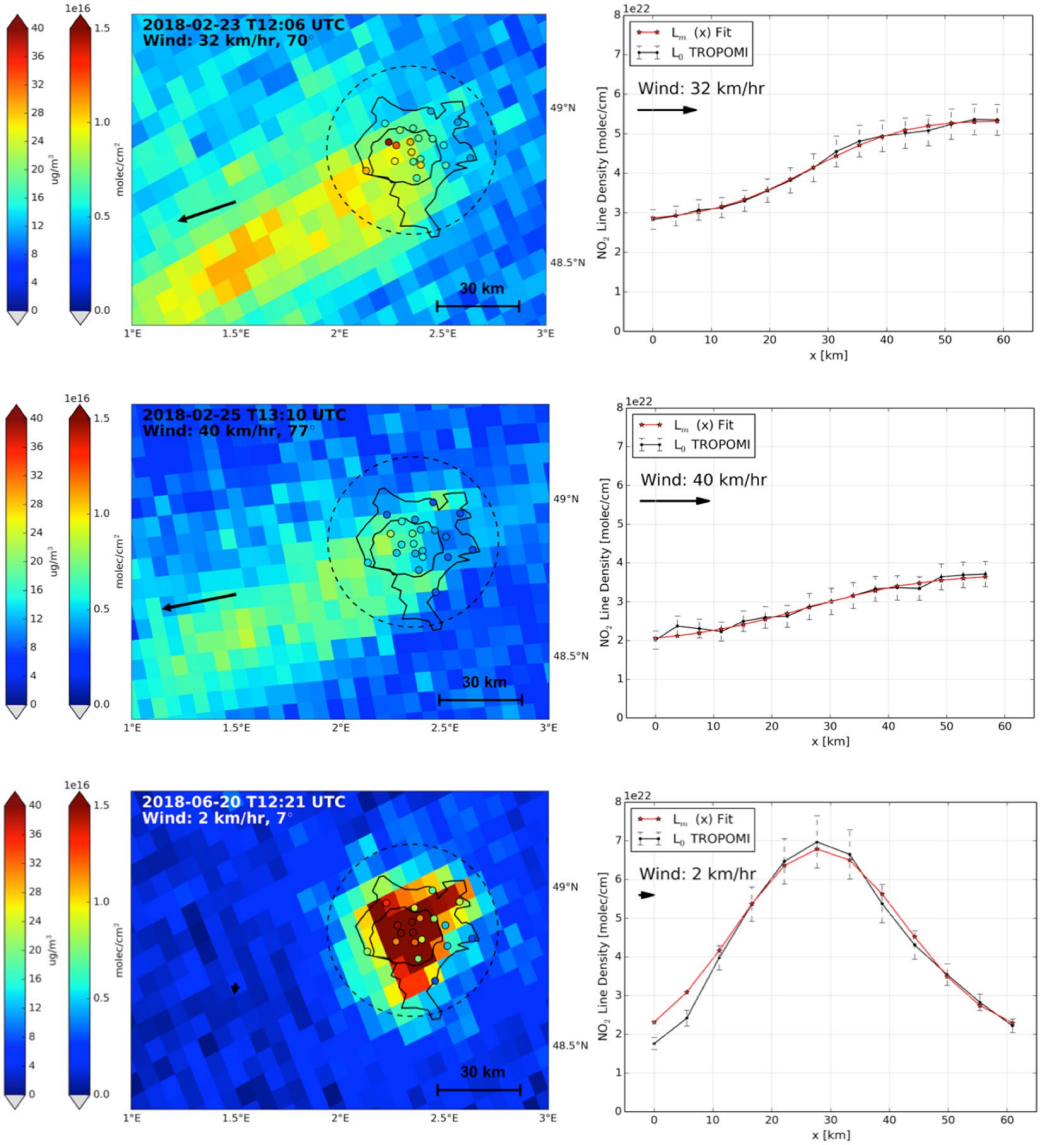
(**a**) Tropospheric NO_2_ columns over Paris on Friday 23 February 2018, (**c**) Sunday 25 February 2018, and (**e**) Wednesday 20 June 2018 observed by TROPOMI. Boundary layer mean wind speed and wind direction, indicated by the black arrow, are from ECMWF ERA-Interim data. The open circles represent the NO_2_ surface concentration in μg m^−3^ measured at urban background stations within 30 minutes of the TROPOMI overpass time (leftmost colour bar). The Paris A86 ring road is indicated by the inner black line, and the city limits are approximated by the outer black line. The right panels (**b**,**d**,**f**) show the corresponding NO_2_ line densities between the upwind and downwind city limits at 0 and 60 km, with line densities calculated by integrating over 60 km perpendicular to the wind (see Methods and Fig. S2).

Paris is one of the three megacities in Europe, next to London and Istanbul, and one of the strongest isolated hotspots of air pollution in north-western Europe, with 10.5 million inhabitants, and more than 3 million cars entering the city each day. Figure 3 shows tropospheric NO_2_ columns over this region on Friday 23 February 2018 measured by TROPOMI. The spatial distribution shows an increase in NO_2_ columns from the northeast towards the southwest over Paris, and downwind of the city a plume of enhanced NO_2_ advected towards the southwest, consistent with predominantly north-easterly winds (32 km/h) on that day. NO_2_ surface concentrations measured at 20 stations throughout Paris^19^ suggest a similar increase in surface pollution from the northeast towards the southwest, even though the measurement techniques are very different. Two days later, on Sunday 25 February 2018, the wind (40 km/h) was slightly stronger, but the build-up of NO_2_ over the city was much weaker, a first indication of lower emissions on this weekend day. This analysis suggests that direct attribution of the NO_2_ increase over Paris to the NO_x_ source strength is possible, if the influence of wind speed and NO_x_ loss processes are accounted for.

On days with very low wind speeds, NO_x_ emissions from the city are hardly ventilated, but concentrate over the city instead. The satellite NO_2_ distribution over Paris then closely resembles the underlying emission pattern. Figure 3 (lower panel) shows the distribution on Wednesday 20 June 2018, a day with a high-pressure system centred over Paris. In the hours prior to TROPOMI overpass, a very light wind (0–2 km/hr) was coming from the north^22^. The high NO_2_ columns observed over inner Paris display a clear echo of the underlying spatial distribution of NO_x_ emissions (Fig. S1). The NO_2_ distribution on 20 June 2018 can be used to estimate the NO_x_ emissions, but the photochemical regime is more complex in stagnant conditions, and assumptions on the chemical lifetime of NO_x_ are more critical than in well-ventilated situations: with a wind speed of 30 km/h, it takes 2 hours for an air parcel to cross the city, too short for substantial chemical loss of NO_x_^4,7^. With a wind speed of 5 km/h however, it takes 12 hours, long enough for substantial chemical conversion of NO_2_ into HNO_3_ over the city.

#### Estimating NO_x_ emissions from TROPOMI line densities

By integrating the TROPOMI NO_2_ columns perpendicular to the wind direction over a distance of 60 km, we constructed so-called line densities^17^ (see Methods). Increases in NO_2_ line density along with the wind are directly linked to recent NO_x_ emissions over the metropolitan area, somewhat dampened by photochemical decay. The right panels of Fig. 3 show the NO_2_ line densities for 23 and 25 February, and 20 June 2018 as function of the along-wind distance over Paris. For the windy February days, the line densities show a modest increase of NO_2_ with distance over the north-eastern segment of the city, followed by a steeper increase over and just downwind of the city centre, and a levelling off towards the south-western edge of the city. This pattern is apparent on many days (Fig. S6) and reflects the spatial distribution of emissions within the Paris Metropolitan Area, where most NO_x_ is emitted in the region enveloped by the A86 ring road, and much weaker emissions occur in the outskirts (Fig. S1). On days with high wind speeds, line densities over the city provide a convolved view of the underlying emission pattern, because the wind disperses the recently emitted nitrogen oxides. On 20 June when wind speeds were low, the line density closely resembles the spatial distribution of the NO_x_ emissions at the approximate scale of a TROPOMI pixel.

To determine NO_x_ emissions, we first tried to directly fit the total emission flux and NO_x_ lifetime from the observed NO_2_ build-up on clear-sky days. This approach assumes that NO_x_ emissions are spatially uniform throughout the Paris domain. Although we obtained reasonable total emission fluxes, there were substantial discrepancies between the observed and modeled line densities. We then generated a large ensemble of pre-computed line densities, each a function of wind speed, NO_x_ loss rate constant, strength and now also of the pattern of the NO_x_ emissions (see Methods). We compared each member to the observed line density, to identify the member and its driving parameters that minimizes the differences between the modelled and the observed line density. In the ensemble, we take as prescribed parameters boundary layer average wind speed from ECMWF, and the [NO_2_]:[NO_x_] ratios simulated by the CAMS model^23^ over Paris for the day of interest (see Methods). We allow the NO_x_ loss rate constants and the emission pattern (with 12 cells of ~5 km over Paris) to vary within a predefined range in our ensemble. As a first guess for the NO_x_ loss rate constant, we use boundary layer mean [OH] from CAMS within a factor 2 given the considerable uncertainty in simulating OH over a large city^7,24,25^ by models (see Fig. S4). The emission pattern is inherently uncertain, so we also allow this to vary. The 1-D TNO-MACC-III NO_x_ emissions pattern^26^ resembles a Gaussian distribution (Fig. S5) and is used as first guess. We then use in our ensemble a range of Gaussian shapes by varying 4 parameters: amplitude (up to factor 3 different), widths (±15%), offsets (factor 2), and centre locations (±5 km). The observed along-wind line densities are described well by the modelled function giving the smallest residuals, illustrated in the right panels of Fig. 3, and the high correlation coefficients between the observations and modelled function (average R^2^ = 0.953), and low unexplained residuals (13%). Because of the TROPOMI overpass of approximately 13:40 hrs, the inferred NO_x_ emissions are generally representative for the hours just prior to the TROPOMI overpass, i.e. noontime^25^. Table 1 summarizes our main results. Total uncertainty in the NO_x_ emissions is mostly driven by the uncertainties in the S5P-TROPOMI NO_2_ columns, and contains non-negligible contributions from uncertainty in wind speed and a priori assumptions on NO_x_ loss rate and emission patterns. We add these contributions in quadrature and estimate an overall emission uncertainty of 36–65% (see Supplementary Material, section 4).

**Table 1:** Paris Metropolitan Area NO_x_ emissions inferred from TROPOMI in 2018, and conditions under which they have been derived.

Day	Time (utc)	Emissions (mol s^−1^)	Wind		NO_x_ lifetime	[NO_x_]:[NO_2_]		Surface temperature	PBL height (m)	R^2^	RMS residuals
			Speed	Direction		CAMS	Eiffel Tower				
Thu 22–02	12:25	110.0 ± 37.4	8.6 m/s	51° (NE)	16.0 hrs	1.83	1.86	3 °C	874	0.990	8%
Fri 23-02	12:06	93.3 ± 31.3	9.0 m/s	70° (ENE)	10.9 hrs	1.72	1.70	2 °C	907	0.996	4%
Sun 25-02	11:29	51.3 ± 16.5	11.0 m/s	77° (E)	9.4 hrs	1.75	1.78	0 °C	798	0.933	15%
Sun 25-02	13:10	74.4 ± 23.9	11.0 m/s	77° (E)	11.4 hrs	1.75	1.86	0 °C	798	0.973	10%
Mon 26-02	12:51	78.7 ± 24.9	12.2 m/s	56° (NE)	10.3 hrs	1.73	1.78	−2 °C	1545	0.925	21%
Tue 17-04	12:18	87.6 ± 32.8	6.4 m/s	197° (S)	1.6 hrs	1.39	1.58	17 °C	1187	0.995	5%
Wed 18-04	12:59	64.5 ± 23.5	6.9 m/s	100° (E)	2.0 hrs	1.19	1.16	23 °C	1251	0.994	5%
Sat 21-04	12:44	38.4 ± 24.1	2.7 m/s	100° (E)	2.9 hrs	1.31	1.28	24 °C	2106	0.970	9%
Sun 22-04	12:25	27.7 ± 9.1	9.7 m/s	230° (SW)	2.7 hrs	1.26	1.39	24 °C	2188	0.840	31%
Tue 24-04	13:29	86.8 ± 36.3	5.0 m/s	231° (SW)	2.8 hrs	1.48	N.A.	15 °C	1292	0.980	9%
Wed 02-05	12:39	65.2 ± 21.9	9.0 m/s	206° (SW)	1.6 hrs	1.18	1.39	14 °C	1499	0.990	7%
Fri 04-05	12:01	50.4 ± 18.6	6.6 m/s	23° (NNE)	2.9 hrs	1.21	1.36	16 °C	1051	0.957	12%
Sat 05-05	11:42	49.2 ± 18.0	6.8 m/s	27° (NNE)	2.9 hrs	1.22	1.24	19 °C	682	0.984	7%
Sat 05-05	13:23	33.5 ± 12.2	6.8 m/s	27° (NNE)	1.8 hrs	1.22	1.11	19 °C	682	0.964	10%
Sun 06-05	13:04	24.8 ± 10.1	5.3 m/s	53° (ENE)	1.9 hrs	1.20	1.31	23 °C	696	0.937	11%
Tue 08-05	12:26	45.6 ± 36.7	2.0 m/s	5° (N)	2.1 hrs	1.23	1.16	24 °C	1699	0.959	10%
Wed 09-05	13:49	36.4 ± 17.3	4.0 m/s	300° (WNW)	2.5 hrs	1.16	1.17	19 °C	775	0.990	7%
Fri 11-05	13:11	48.4 ± 20.9	4.7 m/s	167° (S)	2.2 hrs	1.21	1.44	17 °C	1491	0.982	9%
Thu 17-05	12:58	44.8 ± 18.7	5.0 m/s	12° (N)	2.4 hrs	1.26	1.60	15 °C	844	0.992	6%
Fri 18-05	12:39	40.1 ± 18.0	4.4 m/s	21° (N)	3.2 hrs	1.24	1.72	14 °C	787	0.983	9%
Sat 19-05	12:20	36.4 ± 17.3	4.0 m/s	26° (NNE)	2.4 hrs	1.25	1.31	18 °C	1038	0.903	24%
Wed 20-06	12:20	54.3 ± 57.3	0.5 m/s	7° (N)	3.3 hrs	1.61	1.4	25 °C	959	0.984	11%
Wed 20-06	14:00	75.6 ± 67.9	1.8 m/s	7° (N)	3.9 hrs	1.55	1.4	25 °C	959	0.942	20%
Thu 21-06	12:02	30.5 ± 12.8	5.0 m/s	352° (N)	2.0 hrs	1.30	1.66	20 °C	1139	0.947	15%
Sat 23-06	13:05	17.7 ± 8.3	4.1 m/s	35° (NNE)	1.7 hrs	1.34	1.44	19 °C	1094	0.653	38%
Mon 25-06	12:27	51.7 ± 21.2	5.2 m/s	46° (NE)	1.8 hrs	1.26	1.42	23 °C	1591	0.976	11%
Tue 26-06	12:08	44.8 ± 22.8	3.6 m/s	29° (NNE)	2.1 hrs	1.33	1.41	24 °C	1049	0.949	18%
Tue 26-06	13:50	37.1 ± 18.9	3.6 m/s	29° (NNE)	2.1 hrs	1.33	1.26	24 °C	1049	0.990	8%
Wed 27-06	13:31	49.5 ± 23.2	4.1 m/s	50° (NE)	1.1 hrs	1.25	1.29	26 °C	1360	0.964	11%
Thu 28-06	13:12	41.9 ± 16.1	6.0 m/s	54° (NE)	2.5 hrs	1.50	1.31	26 °C	1967	0.962	10%

Days with two estimates had Paris covered by two successive orbits (orbit 1916 and 1917 on 25 February, 2895 and 2896 on 5 May, 3548 and 3549 on 20 June, 3633 and 3634 on 26 June).

#### Day-to-day variability in NO_x_ emissions

**Figure 4:**
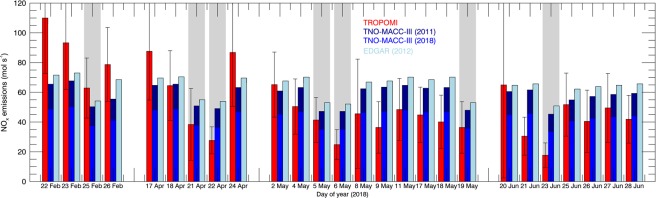
Time series of TROPOMI (red), TNO-MACC-III (2011: dark blue, 2018: blue), and EDGAR (2012) (light blue) NO_x_ emissions integrated over 60 × 60 km^2^ around Paris. The medium blue bars represent projected TNO-MACC-III emissions for 2018 based on reductions of 26% for France between 2011 and 2018 predicted by the EEA^28^. The grey shaded areas indicate weekend days. For days with two TROPOMI emission estimates, the mean is shown. Error bars correspond to 1-sigma uncertainties calculated via error propagation^17,40^ (Supplementary Material).

We compare the TROPOMI NO_x_ emissions for Paris on clear-sky days to emissions from the TNO-MACC-III (2011) and EDGAR^27^ (2012) inventories. The emissions are sampled for the same month, day of the week, and 12:00 hrs local time (see Methods) as the TROPOMI estimates. It is well known that Parisian NO_x_ emissions are dominated by traffic and heating^7,26^. As a result of tightening emission standards (Euro-IV, V, and VI norms) and the more modern vehicle fleet in 2018 compared to 2011^26,28^, we anticipate our TROPOMI estimates to be lower than the inventory estimates for 2011–2012. Figure 4 shows that the TROPOMI emissions for 2018 are (on average) 5–15% lower than the inventory estimates for 2011–2012, but still a long way from the emissions projected for 2018 based on country-specific reductions for France (−26% relative to 2011)^28^.

In February 2018, a particularly cold month (4 °C colder than normal), TROPOMI emissions are higher than those from the inventories. In April-June 2018, surface temperatures were above the 18 °C threshold commonly assumed for turning on heating, and TROPOMI emissions are below those predicted by the inventories. This points to an underestimation of residential heating emissions in cold February, and an overestimate in the warm spring months, associated with the climatological rather than real-time activity factors for residential heating contributions assumed in the inventories. The TROPOMI emissions furthermore show a more pronounced weekend reduction (35%) than the inventory (21%). This is consistent with a smaller role for residential heating in April-June 2018 than predicted by the inventory, and suggests that traffic emissions with pronounced weekend reductions dominate overall NO_x_ emissions in April-June. TROPOMI measurements are thus useful to not only evaluate absolute emissions, but also their temporal disaggregation into monthly, weekly, and diurnal estimates. These come with substantial uncertainties as they are based on behavioural patterns that are assumed to be the same across Europe. One example is that our emission estimates for Fridays are generally lower that those for other weekdays, in contrast to the predictions from the inventory, where Friday emissions are the highest of the week (Fig. S3).

The noontime NO_x_ lifetimes from our method merely represent an improvement to prior, uncertain knowledge on OH concentrations from the CAMS model, constrained via the observed line densities. The lifetimes are about 11 hours in winter and 1–4 hours in spring (with typical uncertainties of 50%), corresponding to mean OH concentrations between 1 and 12 × 10^6^ molec./cm^3^, consistent with other estimates^7,17,24,25^. TROPOMI provides information on the sub-urban distribution of emissions in Paris. The spatial variability in our inferred NO_x_ emissions is similar to the a priori distribution from TNO-MACC-III (Fig. S5).

## Discussion

We show that the new TROPOMI NO_2_ measurements provide good quality information at a resolution unprecedented for satellite remote sensing. The new satellite measurements allow detailed day-to-day monitoring of NO_x_ emissions from Paris for cloudless spells. Our results indicate that NO_x_ emissions in 2018 are only 5–15% below inventory estimates for 2011–2012, falling short of anticipated reductions based on predicted improvements in technology and policies. This is most relevant on cold weekdays, when TROPOMI detects very high emissions compared to the inventories, pointing at strong contributions from the residential heating sector.

With the global coverage of TROPOMI measurements, emissions can be estimated for other major sources around the world in a consistent manner, as long as accurate knowledge of wind speed is available. The method is only weakly sensitive to assumptions on NO_x_ chemical lifetime for days with modest to strong wind speeds, when NO_x_ loss over the city is dominated by outflow. For days with stagnant air and large-scale subsidence the observed NO_2_ patterns provide a direct echo of the NO_x_ emission pattern over Paris. We conclude that the data and methodology presented here demonstrate the potential of TROPOMI and follow-up geostationary sensors to monitor emissions in ever greater spatial and temporal detail, not just for nitrogen oxides, but also for other air pollutants such as carbon monoxide, sulfur dioxide, and formaldehyde. TROPOMI data holds a strong promise for the detection of emissions also from smaller cities and point sources, and is particularly interesting for still uncharted sources.

## Methods

### TROPOMI sensor

The TROPOspheric Monitoring Instrument^12^ (TROPOMI) on board of the Copernicus Sentinel 5 Precursor (S5P) satellite was launched on 13 October 2017. The first 6 months of the mission were used for special observations to commission the satellite and the ground processing systems; the operational phase started in April 2018. The instrument measures the top of the atmosphere solar radiation reflected by and radiated from the Earth between 270–500 nm and 675–775 nm, and in the shortwave infrared. The instrument images a strip of the Earth on a 2-D detector for a period of 1 second during which the satellite moves by about 7 km. The two dimensions of the detector allow to simultaneously measure 450 spectra over the entire 2600 km strip, corresponding to a spatial resolution of the 7 km (along) × 3.5 km (across) at nadir. The equator crossing time is near 13:30 local solar time, which results occasionally in two overpasses over Paris on the same day, with some 100 minutes in between measurements. We use level-2 NO_2_ tropospheric column data (version 1.0.2) processed by KNMI and DLR for February-June 2018^14^. Data from 30 April 2018 onwards in freely available via https://s5phub.copernicus.eu/.

### NO_2_ retrieval algorithm

The NO_2_ columns are retrieved with a 3-step procedure performed for each measured level-1b spectrum as described in the Algorithm Theoretical Baseline Document^14^. In the first step, NO_2_ slant column densities, defined as the integrated amount of NO_2_ along the average photon path from the Sun through the atmosphere back to the sensor, are obtained from the radiance and irradiance spectra using the Differential Optical Absorption Spectroscopy technique in the 405–465 nm window where NO_2_ has prominent spectral features. Then, the slant column is separated into a stratospheric and tropospheric part based on information from a data assimilation system. Finally, the tropospheric slant columns are converted into tropospheric vertical column densities by application of a tropospheric air mass factor (AMF) based on a look-up table of altitude-dependent AMFs and actual information on surface and cloud characteristics and on the vertical distribution of NO_2_ predicted by the TM5-MP model on a 1° × 1° grid^29^.

The TROPOMI retrieval builds on principles used in the DOMINO v2 approach^30^, but includes many retrieval improvements proposed within the European Union Quality Assurance for Essential Climate Variables (QA4ECV) project^16,31^. These include an improved wavelength calibration and the inclusion of O_2_-O_2_ and liquid water in the DOAS fitting model^32,33^. In combination with the high signal-to-noise ratio of TROPOMI, this leads to lower uncertainties in the slant columns (0.5–0.6 × 10^15^ molec.cm^−2^) compared to OMI. The data assimilation approach to estimate the stratospheric NO_2_ columns is based on the TM5-MP model and operates on a 1° × 1° grid^29^. Stratospheric NO_2_ in TM5-MP (free running mode) is driven by nudging to satellite-observed climatological HNO_3_:O_3_ ratios (from ODIN and HALOE) and 3-hourly ECMWF analysed and forecast meteorological fields. The TM5-MP simulations are updated every 30 minutes based on TROPOMI slant columns available in that timestep, and the forecast NO_2_ field is then used to estimate the stratospheric NO_2_ column. A preliminary comparison with ground-based NDACC SAOZ measurements suggests agreement to within 10% (or 0.3 × 10^15^ molec.cm^−2^) between the TROPOMI and SAOZ stratospheric NO_2_ columns^34^. The air mass factor that converts the slant column density to vertical column density is calculated using the radiative transfer model Doubling-Adding code KNMI. The AMF includes a correction factor to account for atmosphere’s sphericity calculated with the 3D model McArtim^31^. This correction affects mainly the stratospheric AMFs that are highly relevant in the stratospheric NO_2_ estimation, and results in lower stratospheric NO_2_ columns especially for extreme solar zenith angles in the winter hemisphere^16^. The a priori NO_2_ vertical profiles from TM5-MP have an improved spatial resolution with respect to DOMINO.

### TROPOMI NO_2_ data filtering

We use tropospheric NO_2_ columns measured from orbits with cloud radiance fractions less than 0.5, corresponding to geometric cloud fractions of up to 0.2, over Paris, as recommended in the TROPOMI ATBD^14^. Experience with previous satellite NO_2_ observations showed that measurements with the lowest effective cloud fractions compare most favourably with independent measurements^35^, and are of the highest quality^31^. Following this criterion rendered a total of 36 orbits (obtained on 29 different days) with a mostly unobstructed view on Paris, corresponding to a retention rate of approximately 25% in the February-June 2018 period.

Arguably the largest source of uncertainty in the satellite retrievals is the computation of the tropospheric AMF^16,31^. We evaluated the influence of cloud parameters (effective cloud fraction and cloud pressure) on the AMF patterns, and found that in some situations the rather course-gridded surface albedo climatology used in the FRESCO+ cloud pressure retrieval, led to spurious jumps in the tropospheric air mass factors for some days, which were absent when evaluating the tropospheric slant column divided by the geometrical air mass factor. Based on these tests, we rejected a number of orbits from further analysis: 24 February (orbits 1902 and 1903). This was also a day with relatively high cloud radiance fractions over Paris. We also rejected data from 20 April 2018, as on this day various pixels right in the middle of the pollution build-up (13 out of 126) were classified as non-valid over Paris. A day with rapidly changing wind direction (19 April) was not considered either in our analysis. There were strong indications for winds shifting direction and speed in the hours just before the TROPOMI overpass time^22^ of 13:22 UTC.

Our validation exercise over Paris suggests that TROPOMI NO_2_ columns have a low bias with a multiplicative component of 25% (Fig. 2). This is in line with other validation activities that suggest that TROPOMI NO_2_ columns are on average some 20–40% lower than co-located NO_2_ columns measured with independent ground-based measurements^21,34^. This could be indicative of a low bias in the satellite retrievals or a high bias in the ground-based measurements, but could also indicate differences in spatial representativeness between the ground-based and satellite measurements. An experiment in which we recalculated TROPOMI air mass factors by replacing a priori assumptions on the NO_2_ vertical distribution (from TM5-MP) by profile shapes simulated with a high-resolution model, shows higher NO_2_ columns, and suggests stronger increases of NO_2_ along with the wind from TROPOMI.

### Line densities

For each day, the tropospheric NO_2_ columns are converted into one-dimensional line densities along the wind direction over Paris. We do so by gridding the original TROPOMI field to a 0.05° × 0.05° grid rotated towards the mean wind direction in the boundary layer (Fig. S2). We then integrate this rotated field over a 60 km interval perpendicular to the wind direction, in units of molecules cm^−1^. The 60 km interval is motivated by the spatial contours of Paris and the horizontal distribution of bottom-up emissions that suggest that the entire Paris metropolitan area is enveloped in all directions within a radius of 30 km (Fig. S1), and so avoids interference from other sources. We focus on the pollution build-up over the city along with the wind. Our line densities thus start 30 km upwind of the Paris city centre (*x* = 0 km), and end 30 km downwind (*x* = 60 km). This ensures that they capture the full extent of the accumulation of NO_2_ over the emitting metropolitan area.

### Boundary layer information on wind and chemistry

We calculate mean boundary layer wind speed at 12 UTC (13:00 hrs local time in Paris) from ECMWF data. The wind data is taken from the 6-hourly data ECMWF ERA-Interim reanalysis^18,36^. The original horizontal resolution of this dataset is about 80 km, we use a re-gridded version at 0.125° × 0125°. At the TROPOMI overpass time, the boundary layer is usually well mixed, so the boundary layer average wind speed and wind direction is a good representation of the transport of pollutants within the Paris dome. We verified that the NO_2_-weighted mean boundary layer wind speed did not differ significantly from the unweighted boundary layer mean wind speed.

The a priori NO_x_ lifetime is expressed as ${\tau }_{N{O}_{x}}=\frac{[{{\rm{NO}}}_{{\rm{x}}}]/[{{\rm{NO}}}_{2}]}{k[{\rm{OH}}][M]}$, with [NO_x_], [NO_2_], and [OH] the boundary layer mean (initial) concentrations simulated by CAMS at 12 UTC over Paris, and *k*[M] the high-pressure, high-temperature reaction rate constant for oxidation of NO_2_ (2.6 × 10^−11^ cm^3^ molecule^−1^ s^−1^). The CAMS global near-real time atmospheric composition reanalysis provides 3-hourly information of atmospheric composition, with a horizontal resolution of 40 km. The CAMS system uses satellite observations of atmospheric composition in its 4-D variational data assimilation system^23^, together with the Integrated Forecast System for atmospheric composition (C-IFS) from ECMWF^37^. CAMS (initial guess) OH simulations over Paris have been evaluated with OH from the CLASS mixed-layer model^38^ and the high-resolution (6.7 km) WRF-Chem model in the Supplemental Material.

### Simulating line densities with a superposition model

We constructed a simple model that simulates the NO_2_ line density as a function of the along-wind distance *x* exclusively over Paris. This model simulates the build-up of NO_2_ in the air column as a superposition of line densities caused by the emissions in cell *i*. The prior emission in these cells is from the TNO-MACC-III NO_x_ emissions, integrated cross-track over the full 60 km width perpendicular to the wind over Paris (unit molecules NO_x_ cm^−1^ s^−1^)). For each ~5 km long cell *i* between 0 km ≤ *x* ≤ 60 km the contribution to the line density downwind of the cell is calculated using a simple column model^4^:

2 $$\begin{array}{cc}{N}_{i}(x)=\frac{E({x}_{i})}{k}(1-{e}^{-k(x-{x}_{i})/u})\frac{[{{\rm{N}}{\rm{O}}}_{2}]}{[{{\rm{N}}{\rm{O}}}_{{\rm{x}}}]} & for\,x\ge {x}_{i}\\ {N}_{i}(x)=0 & for\,x < {x}_{i}\end{array}$$

where *N*_*i*_*(x*) represents the contribution from *E(x*_*i*_*)*, the NO_x_ emissions from cell *x*_*i*_ alone, to the overall line density, *k* is the loss rate constant of NO_x_ for daytime conversion to nitric acid ($k=\frac{k^{\prime} [{\rm{OH}}][{\rm{M}}]}{([{{\rm{NO}}}_{{\rm{x}}}]/[{{\rm{NO}}}_{2}])}$), and *u* is the vertically averaged boundary layer wind speed. In Eq. (2), scaling with the vertically averaged proportion of [NO_2_]:[NO_x_] mixing ratios is required to express the line densities in terms of NO_2_. The superposition accounts for the spatially varying emission rates (*E(x)*) in the urban area and reads:

3 $$N(x)=\mathop{\sum }\limits_{i=1}^{n}{N}_{i}(x)$$

with *N(x)* describing the build-up of NO_2_ in molecules cm^−1^ along with the wind exclusively over the Paris Metropolitan Area. The build-up reflects the underlying emission strength and pattern *E(x)* and is influenced by the first order chemical loss and wind speed over the city. The background value at the upwind end of the city (*x* = 0 km) is assumed to be representative for and constant over the entire city, so that the increase between 0 and 60 km is only attributed to emissions from the city.

### Daily NO_x_ emissions over Paris from the TNO-MACC-III inventory

To obtain the emission for a particular day at 12:00 hrs (just before the TROPOMI overpass time), the 24-hour mean emissions for Paris were first integrated over a 60 × 60 km^2^ area around the city centre, and then scaled by specific monthly, weekly, and diurnal factors from the TNO-inventory. Fig. S3 illustrates the temporal scaling factors from the TNO-MACC-III inventory, with the highest emissions in February-March, and lowest in June. The weekly cycle in emissions peaks on Friday, and has some 21% lower emissions on weekend days. The diurnal cycle indicates that emissions at 12:00 hrs are 20–30% higher than the 24-hour mean. These factors from a TNO report^39^ have a high uncertainty embedded. For instance, the diurnal cycle in emissions from road transport is based on traffic intensity time series from 1985 to 1998 in the Netherlands. It seems plausible that not only traffic intensity and emission abatement in cars decreased in the past decades, but also that the temporal variation in Paris is different than in the Netherlands.

## Supplementary information

Supplementary Material
