# Supplementary material for: Quantification of nitrogen oxides emissions from build-up of pollution over Paris with TROPOMI
Source: Sci Rep. 2019 Dec 27;9:20033. doi: 10.1038/s41598-019-56428-5 (PMC6934826; doi:10.1038/s41598-019-56428-5)
Supplement: Supplementary file 1 — Supplementary Material [file 41598_2019_56428_MOESM1_ESM.docx]

**Supplementary material for**

**Quantification of nitrogen oxides emissions from build-up of pollution over Paris with TROPOMI**

A. Lorente^1^, K. F. Boersma^1,2^, H. J. Eskes^2^, J. P. Veefkind^2,3^, J. H. G. M. van Geffen^2^, M. B. de Zeeuw^1^, H. Denier van der Gon^4^, S. Beirle^5^, and M. C. Krol^1^

Version 3.0, 7 November 2019

^1^Wageningen University, Environmental Sciences Group, Wageningen, The Netherlands

^2^Royal Netherlands Meteorological Institute, R&D Satellite Observations, De Bilt, The Netherlands

^3^Delft University of Technology, Delft, The Netherlands

^4^TNO, Department of Climate, Air and Sustainability, Utrecht, The Netherlands

^5^Max-Planck-Institut für Chemie, Mainz, Germany

**1. Line densities over Paris**

For each mostly clear-sky day, we calculated maps of NO_2_ tropospheric columns from S5P-TROPOMI observations. We then rotated the maps such that they align with the prevailing ECMWF boundary layer wind direction over Paris. Line densities *L*(*x*) (as function of distance *x* from the point source in wind direction) are determined by spatial integration over a 60 km interval in across-wind direction. The 60 km interval is motivated by the spatial contours of Paris and the horizontal distribution of bottom-up emissions that both suggest that the entire Paris metropolitan area is enveloped in all directions within a radius of 30 km (Figure S1).

| 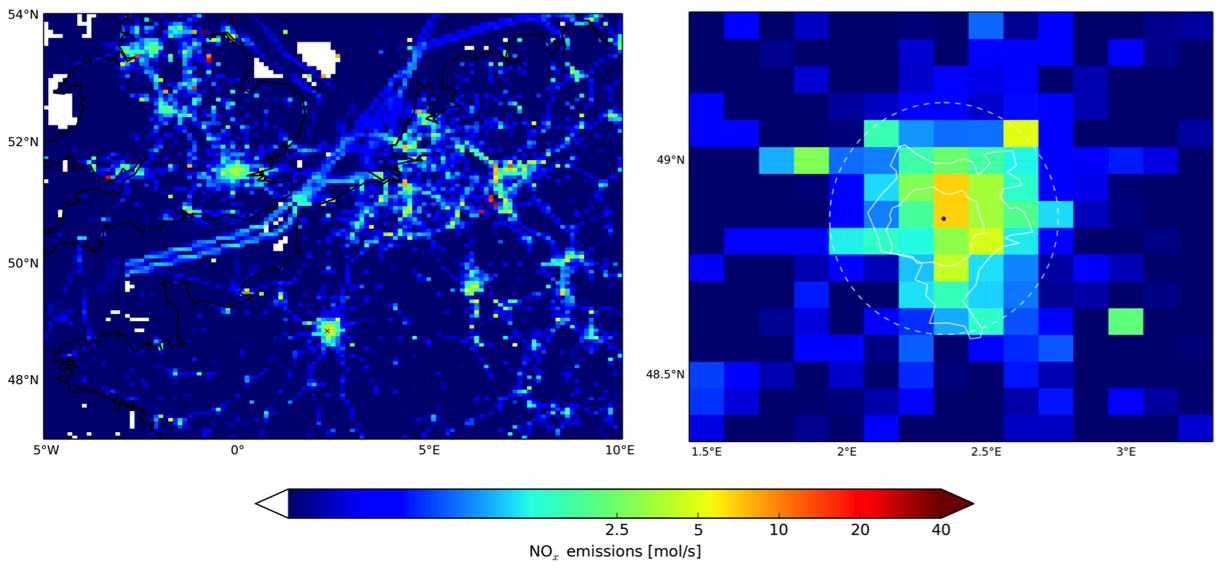 |
| --- |
| **Figure S1**. Upper panels: NO_x_ emission strength (mol s^-1^) over the Paris Metropolitan area from the TNO-MACC-III emission inventory for 2011; original resolution 1/8°×1/16° (lon × lat). In the upper right panel, the location of the A86 ‘Super-périphérique Parisien’ highway and geographical contours of the entire metropolitan area (including Sarcelles, Taverny, Montesson, Versailles, Palaiseau, Bretigny, Villiers sur-Marne, Torcy, Mitry-Mory, and Paris Charles-de-Gaulle airport) are indicated as white solid lines. The 30 km radius envelope is indicated as a dashed white line. |

We focus on the pollution build-up of NO_2_ in a column of air that is advected over the city. Our line densities thus start 30 km upwind of the Paris city centre, and end 30 km downwind. This ensures that line densities capture the full extent of the accumulation of NO_2_ over the metropolitan area. Figure S2 below illustrates our method to calculate the line densities, for Friday 4 May 2018.

| *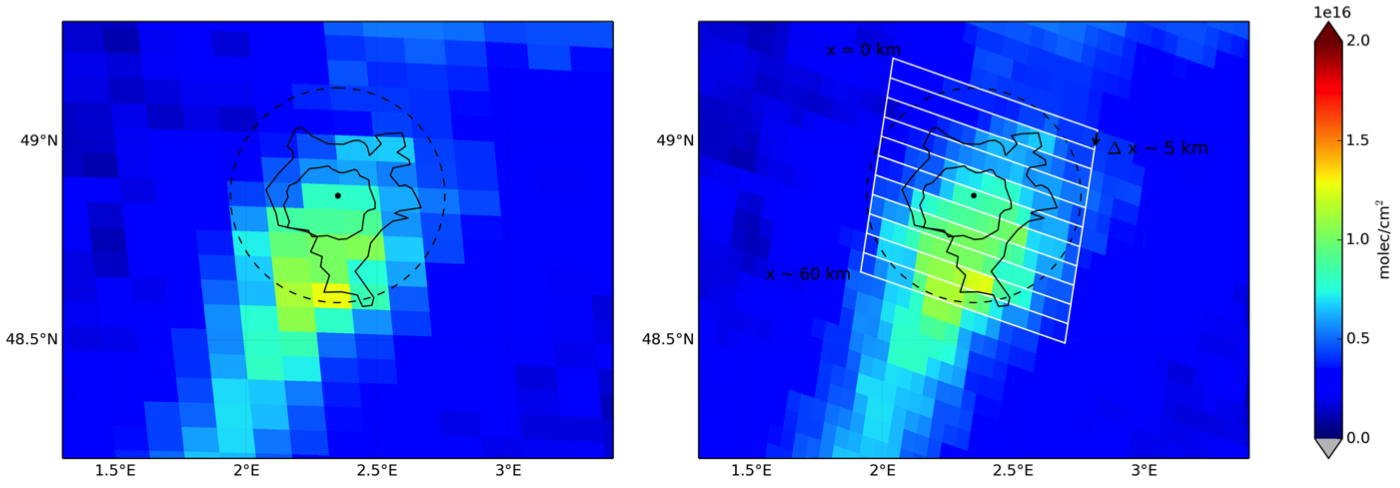* | *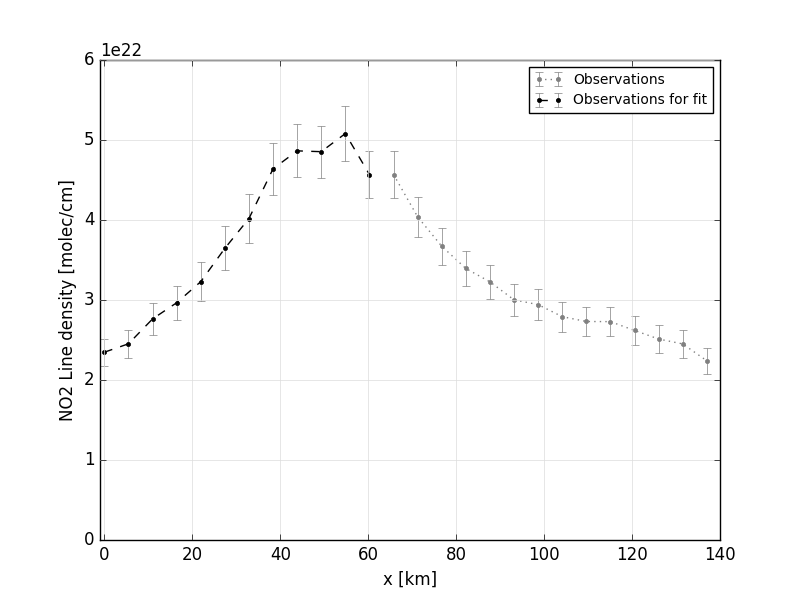* |
| --- | --- |
| **Figure S2.** Left and middle panel: tropospheric NO_2_ vertical column on 4^th^ May 2018 from S5P- TROPOMI, S5P- TROPOMI rotated towards North-Northeast wind direction. Right panel: corresponding NO_2_ line density from 0 km (upwind) to 60 km (downwind) over Paris (black dashed line) with further downwind line density decay up to 140 km in light grey. | |

**2. Temporal variation in NO_x_ emissions**

To account for temporal variations and allow a fair comparison between the inferred S5P-TROPOMI NO_x_ emissions (valid for approximately 12:00 hrs local time) and the NO_x_ emissions predicted by the TNO-MACC-III inventory, we used the emission scaling factors over Paris provided in the TNO-MACC-III inventory [Kuenen et al., 2014]. Figure S3 illustrates the scaling factors that relate the 24-hour mean emissions in the inventory to the emission strength at 12:00 hrs for all the days of the week, and for different months. According to the inventory, weekend reductions are ±30% in winter, and ±20% in summer, and emissions are some 10% lower in the summer months than in winter as a consequence of a smaller contribution from residential heating in the warm season.

| 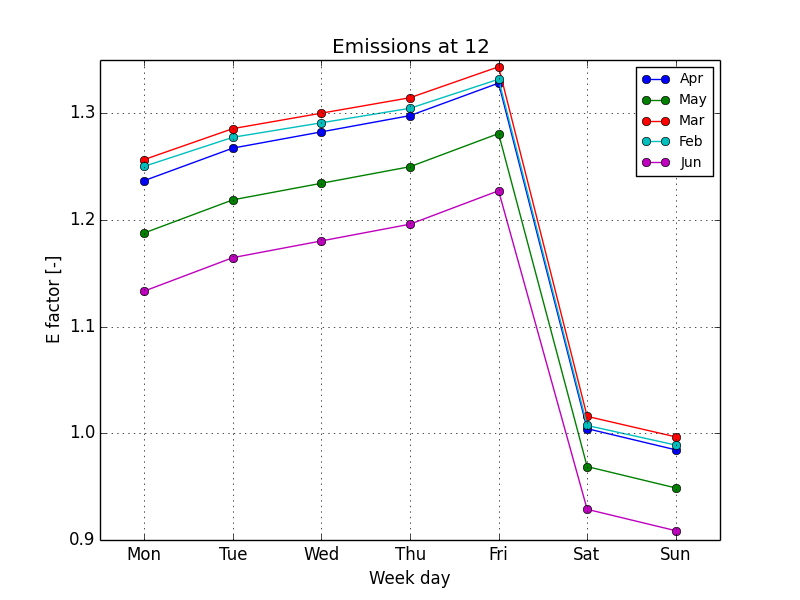 |
| --- |
| **Figure S3.** Emission scaling factors for 12:00 hrs local time over Paris, France, as provided by the TNO-MACC-III inventory. A value of 1.0 corresponds to the 24-hr mean (for the year 2011) NO_x_ emissions provided in the inventory. |

**3. Modeled boundary layer mean OH concentrations**

Because it is difficult for coarse-resolution chemistry transport models to reproduce OH concentrations (Ren et al. [2003]; Valin et al. [2013]), especially over strong source regions such as Paris, we allow in our inversion for a range of ±50% on the boundary layer mean OH concentrations around the initial values predicted by CAMS (40×40 km^2^) over the city. This range was motivated by a comparison of OH simulations by the 3-D CTM CAMS and 0-D CLASS model [Vilà-Guerau de Arellano et al., 2015] showing that OH levels from the latter are some 50% lower (Figure S4) on most days in June. The CLASS box model was forced with (1) initial conditions; (2) emissions; and (3) a chemical scheme, to reproduce the O_3_ and NO_x_ values observed at the 3^rd^ floor of the Eiffel Tower. Further tests with the WRF-Chem model [Visser et al., 2019] with a zoom-in resolution of 6.7×6.7 km^2^ over northwestern Europe, indicate that boundary layer mean OH (averaged over 40×40 km^2^) from WRF-Chem gives comparable concentrations as CAMS over Paris (Supplementary Table S1). WRF-Chem OH concentrations for the single model cell over the city centre (Eiffel Tower) are 20% lower compared to CAMS and 24% lower compared to WRF-Chem at the 40×40 km^2^ domain, well within the 50% uncertainty range of the CAMS, and reflecting modest spatial variability in the simulated OH values.

For all days, we consider the initial OH value simulated by CAMS as the starting point for inversions. For one particular day, 20 June 2018, this led to a poor fitting result. Wednesday 20 June 2018 was a day with extremely low wind speed and a boundary layer reaching an altitude of only 950 m over Paris. This is associated with a high-pressure system and large-scale subsidence, capping the boundary layer. Indeed, the NO_2_ column at the city centre exceeded 2.5×10^16^ molec. cm^-2^ on this day, and NO_2_ concentrations measured at the top of the Eiffel Tower (3^rd^ floor, 300 m altitude) just before the TROPOMI overpass were very high (40 μg/m^3^ compared to 5-15 μg/m^3^ on other June days [AirParif, 2018]), and the boundary layer was not as deep as on other June days. We evaluated this particular situation with the CLASS mixed-layer model, again constrained by measured concentrations of O_3_, NO, NO_2_ and boundary layer height. The CLASS model simulations suggest that daytime OH was indeed lower by a factor 3-5 on 20 June than in the period between 21 and 28 June, when wind speeds and boundary layer heights were much higher. Also WRF-Chem at 6.7 km simulates much lower OH (280 ppq) than CAMS (560 ppq) on 20 June (Supplementary Table S1). Figure S4 shows that the CAMS OH simulations correspond to those from CLASS within a factor 2 between 21 and 28 June, but that CAMS does not capture the reduced OH levels simulated by CLASS for the stagnant situation on 20 June. This can be attributed to fast dispersion of NO_x_ in that model over the ~40 km × 40 km grid boxes, promoting the formation of O_3_ and OH, compared to the high-NO_x_ regime indicated by observations. In contrast, CLASS, constrained by observed NO, NO_2_, and O_3_ concentrations from the Eiffel Tower and boundary layer height from ECMWF, and also WRF-Chem, suggest that OH levels are actually suppressed by the high levels of pollutants (NO_x_, VOCs) that accumulate over Paris on this day without any ventilation and a relatively shallow boundary layer. We therefore reduced the initial boundary layer mean OH concentration from CAMS (15×10^6^ molec. cm^-3^) by a factor of 4 to obtain a more plausible first guess of the NO_x_ loss rate constant on this day that is then in between the CLASS and WRF-Chem values for this day. The inferred emissions from two successive orbits are then on the same order of magnitude as on other June days, the inferred NO_x_ lifetime is somewhat longer, and the quality of the fit improved. Both estimates come with considerable uncertainties because of the low wind speed (Table 1).

| 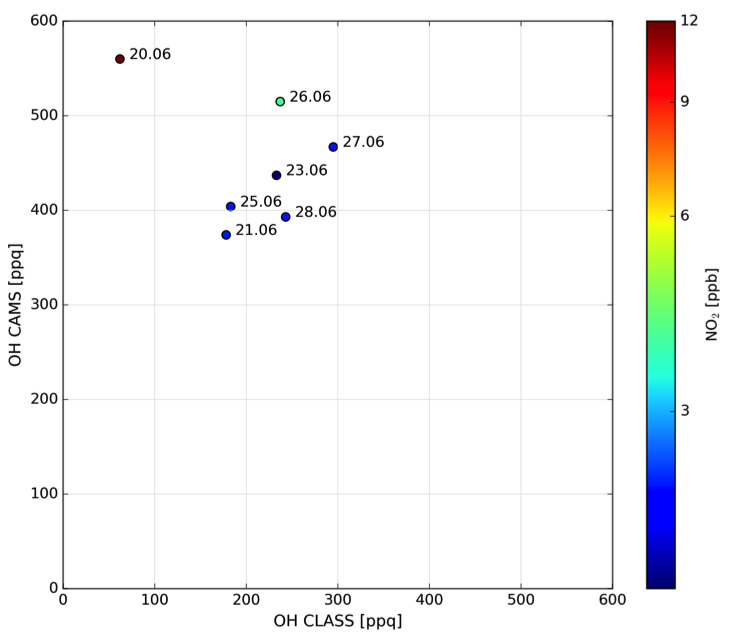 |
| --- |
| **Figure S4.** Scatterplot of OH simulated by the 40×40 km^2^ CAMS CTM ensemble mean and boundary layer CLASS model at the TROPOMI overpass time (12:00-14:00 hrs; constrained by by measurements from AirParif, Eiffel Tower station, 3^rd^ etage). The corresponding NO_2_ concentrations observed at the Eiffel Tower are colour-coded.  **Supplementary Table S1.** Boundary-layer (average) OH concentrations simulated over Paris simulated by different model representations.   \| **Date** \| **CAMS OH (ppq)**  **(40×40 km^2^)** \| **WRF-Chem (ppq) (40×40 km^2^)** \| **WRF-Chem (ppq)**  **(6.7×6.7 km^2^)** \| **CLASS OH (ppq)**  **(Eiffel Tower)** \| \| --- \| --- \| --- \| --- \| --- \| \| 20 June 2019 \| 560 \| 343 \| 280 \| 60 \| \| 21 June 2019 \| 374 \| 600 \| 601 \| 178 \| \| 23 June 2019 \| 437 \| 429 \| 278 \| 233 \| \| 25 June 2019 \| 404 \| 499 \| 319 \| 183 \| |

**4. Uncertainties**

The S5P-TROPOMI NO_x_ emissions derived from S5P-TROPOMI are inherently uncertain. Here we discuss and quantify the main contributions. Our estimated emissions are directly affected by the accuracy of the S5P-TROPOMI columns, which is driven by structural uncertainty in the tropospheric air mass factor calculation [Boersma et al., 2004; Lorente et al., 2017] and amounts to ±30%.

The ratio between NO_2_ and NO_x_ concentrations from CAMS over Paris has an uncertainty of ±20%, based on a comparison of CAMS simulations and observations at the Eiffel Tower (Table 1). If we replace CAMS NO_x_-to-NO_2_ ratio’s (geometric mean: 1.36) by those from the Eiffel Tower (geometric mean: 1.43), our emission estimates are reduced by less than 3% on average. We adopt an error of ±20% in the ECMWF boundary layer mean wind speed, consistent with Beirle et al. [2011], and Petetin et al. [2015]. With this error, we found that emissions changed by +20%, when modifying the wind speed by +20%.

Our results are only weakly sensitive to assumptions on the NO_x_ lifetime. Firstly, our method allows for an uncertainty in the boundary layer mean OH concentrations from CAMS of 50%, reflecting the difficulty that models have in capturing OH over a large city. Because the inferred emissions have some dependency on the selected range of possible OH concentrations, we did a test where we used CLASS OH concentrations (with generally lower values, e.g. Figure S4) as our starting point instead of the CAMS values. Results change by less than 15%, thus our method proves to be quite robust for such changes. If CAMS systematically overestimates the boundary layer OH, then emissions will be overestimated.

The a priori emission pattern is important to minimize the differences between the observed and modelled line densities, but its exact shape is of little influence to the overall emissions. We did an experiment where we used one fixed a priori emission pattern as predicted by the TNO-MACC-III inventory, and compared the resulting emissions to the results obtained by optimizing the pattern as described in the manuscript. The emissions are similar to within 10%.

Other uncertainties include wind direction and wind speed changing with height. Our method assumes that the NO_2_ pollution over Paris is advected in a well-defined direction and that the boundary layer average wind speed captures the advection well. In reality there will be differences in wind direction and wind speed with altitude. The effects of wind direction uncertainties on NO_x_ emissions are likely limited, because the 60 km intervals for calculating line densities are large enough to absorb small errors in wind direction. Wind speed variations with height do not lead to large uncertainties in the estimated emissions. As described in the Methods, the NO_2_-weighted mean boundary layer wind speed did not differ significantly from the mean wind speed between the surface and the boundary layer depth, so that the mean boundary layer wind speed can be considered to be representative for the bulk of the NO_2_ in the boundary layer.

Systematic wind variations, for instance a relatively fast change in the prevailing wind direction, or recirculation, may affect the spatial build-up patterns over the city, and thus the fit results. Often this effect is small, and the observed patterns are dominated by the wind conditions close to the time of the satellite measurement. Days with rapid changes in wind direction or wind speed such as 19 April 2018 have been excluded from further analysis (see Methods).

**Supplementary Table S2.** Evaluation of uncertainties in the individual components and how these affect the NO_x_ emission estimates from S5P-TROPOMI.

|  | **Uncertainty** | **Effect on NO_x_ emissions** |
| --- | --- | --- |
| S5P-TROPOMI NO_2_ column | 30% | 30% |
| NO_2_:NO_x_ ratio | 20% | <3% |
| Wind speed | 20% | 20% |
| A priori NO_x_ loss rate | 50% | 15% |
| A priori emission pattern | 20% | 10% |
| Total uncertainties assuming uncorrelated error contributions |  | ±50% |

The total uncertainty in the NO_x_ emissions is mostly driven by the uncertainties in the S5P-TROPOMI NO_2_ columns, and contains non-negligible contributions from uncertainty in wind speed and a priori assumptions on NO_x_ loss rate and emission patterns. We add these contributions in quadrature and estimate an overall emission uncertainty of 36-65%. These estimates are higher for days with low wind speeds, when wind speed and OH are particularly uncertain, such as on 20 June 2018 when we estimate uncertainties of ±100%.

The fit results are robust with respect to changes in the selection of the across-city integration interval, as long as this interval is chosen wide enough (60 km, all of Paris is captured within a 30 km radius). If this interval is chosen more narrowly, then the line density will not cover the full across-wind extent of Paris, so that the emissions would be representative for a smaller part of Paris.

**5. Spatial distribution of the NO_x_ emissions**

Figure S5 compares the (1-D) emission distribution from TROPOMI and TNO-MACC-III over Paris for all week and weekend days. The patterns agree reasonably well, and indicate that the bulk of the Parisian NO_x_ is indeed emitted within a radius of 15 km of the city centre, with weaker contributions from the suburbs.

| **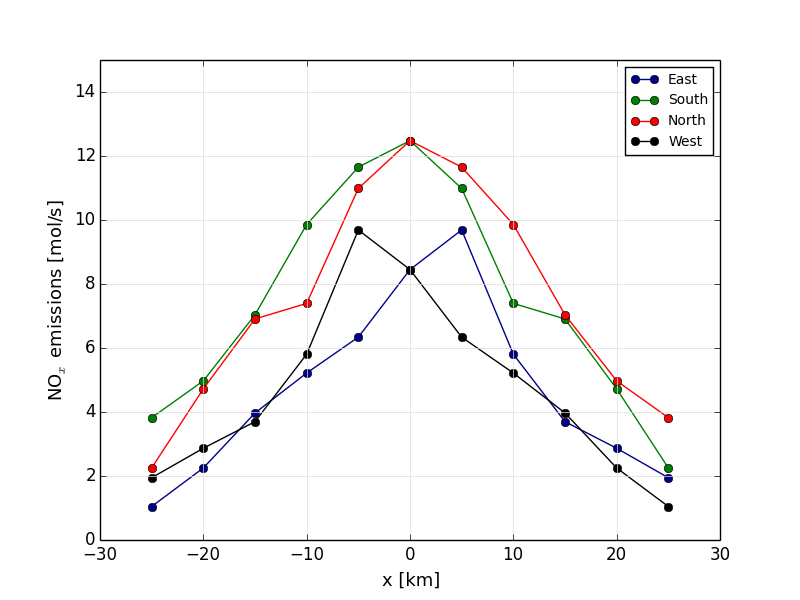** | 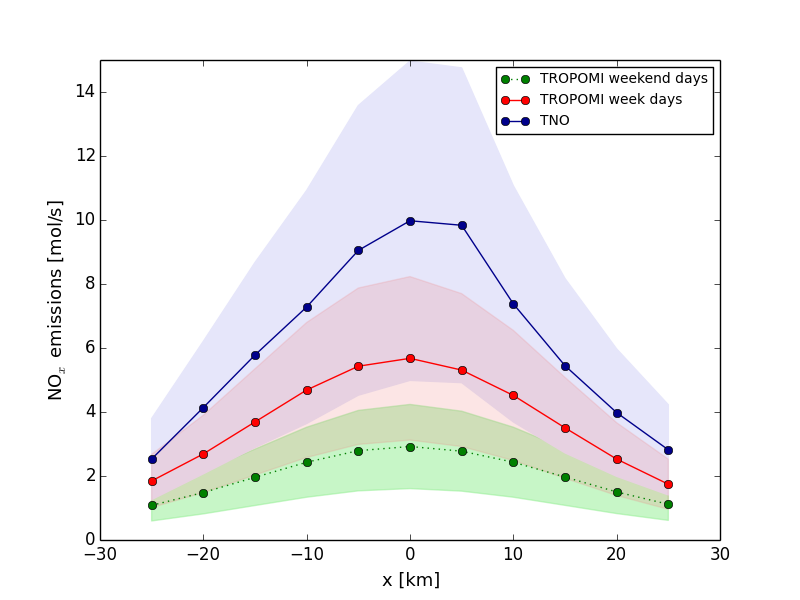 |
| --- | --- |
| **Figure S5.** Left panel: emission ‘line density’, obtained by accumulating TNO-MACC-III NO_x_ emissions across Paris over a 60 km perpendicular to the wind in steps of 5 km. Right panel: average NO_x_ emission line density (mol/s) from TROPOMI weekdays (red) and weekend days (green) and TNO-MACC-III (dark blue) over Paris. The TNO-MACC-III emission line densities were calculated by sampling along the main wind direction, and hold for the samples days in the year 2011. The TROPOMI patterns have not yet been corrected for the low bias discussed in Section S1. | |

**6. S5P-TROPOMI NO_2_ maps, line densities, and model fits for all days**

Figure S6 below shows the S5P-TROPOMI tropospheric NO_2_ columns (left columns) and the observed and fitted NO_2_ line densities (right columns) for all days that were mostly cloud-free and had valid data over Paris in the period February-June 2018.

| **February 2018** | |
| --- | --- |
| 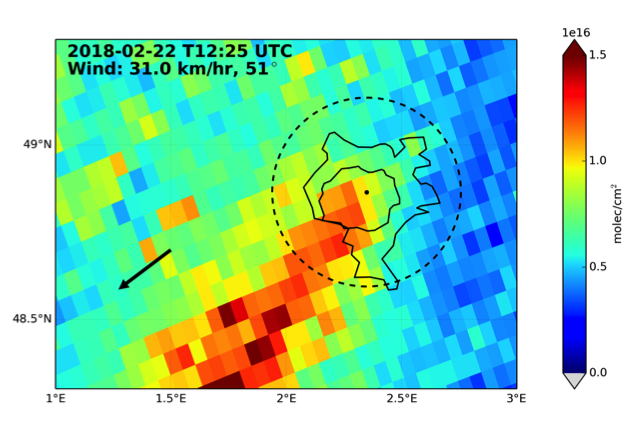 | 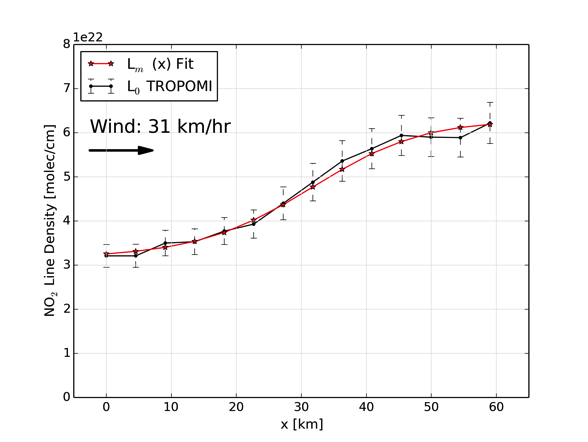 |
| 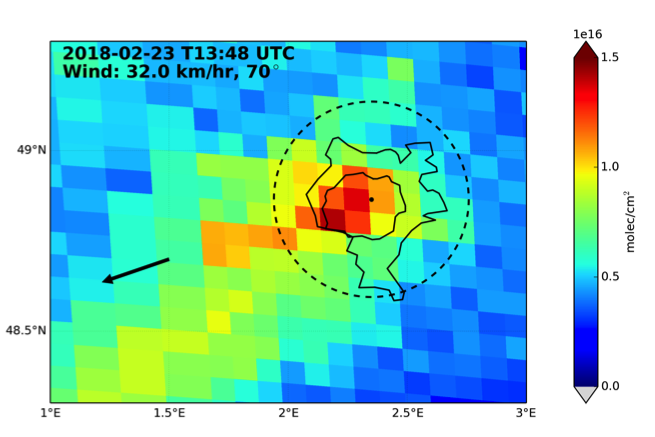 | 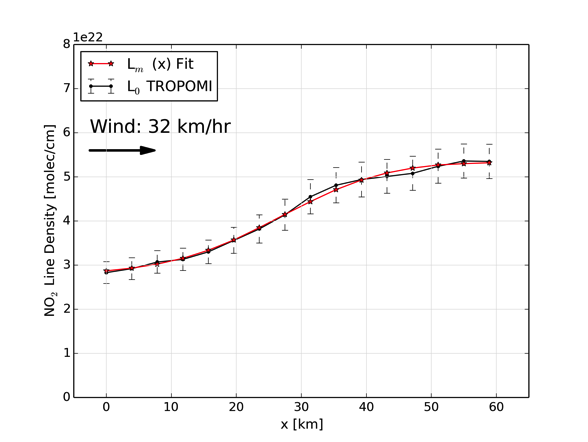 |
| 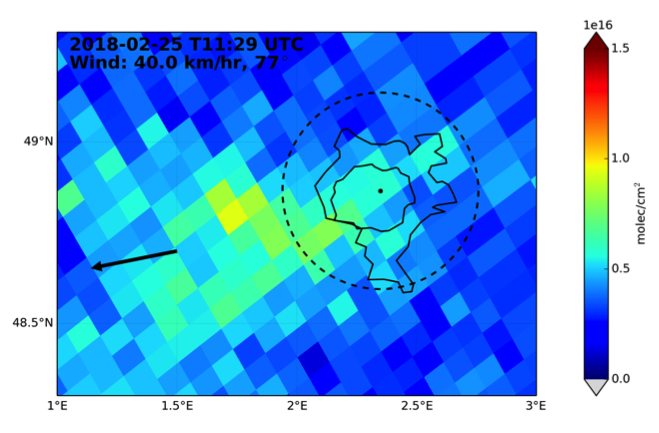 | 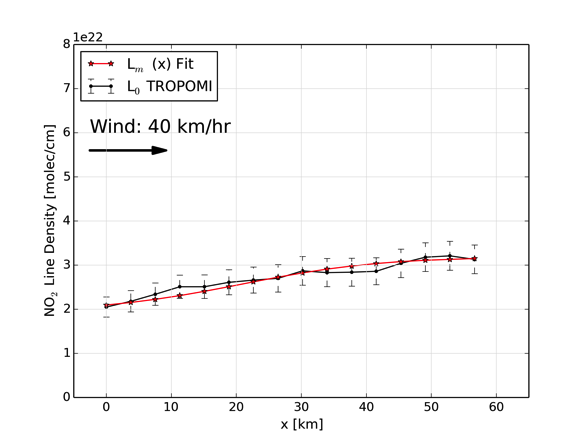 |
| 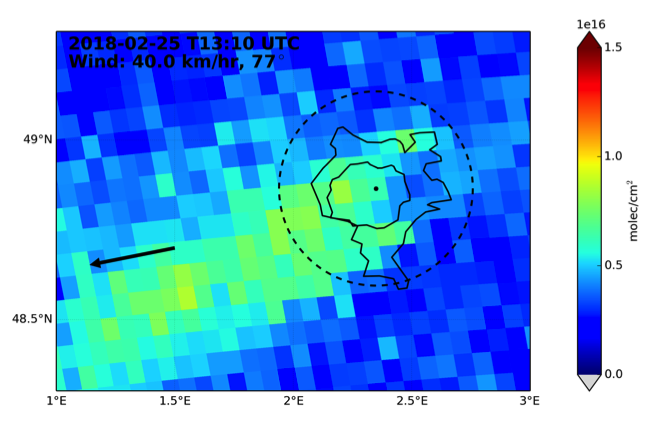 | 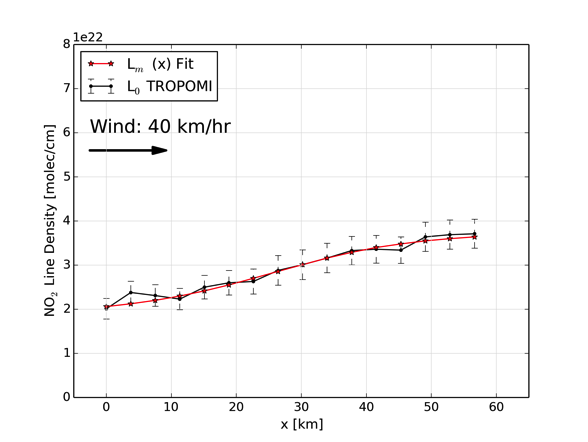 |
| 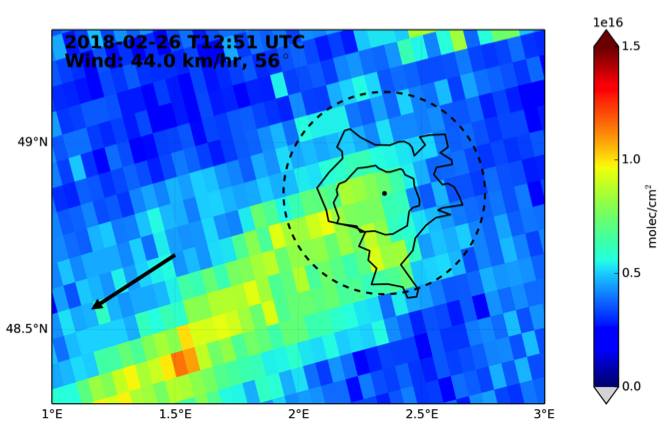 | 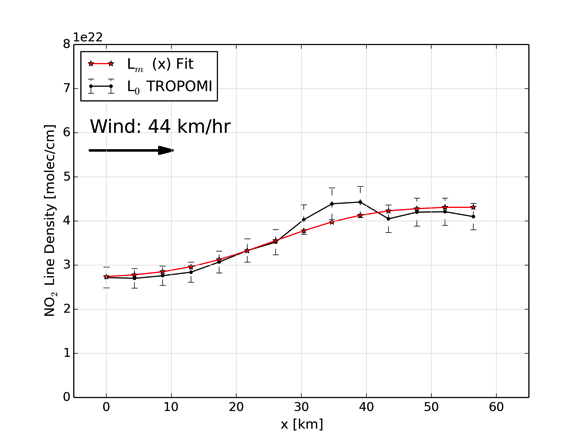 |
| **April 2018** | |
| 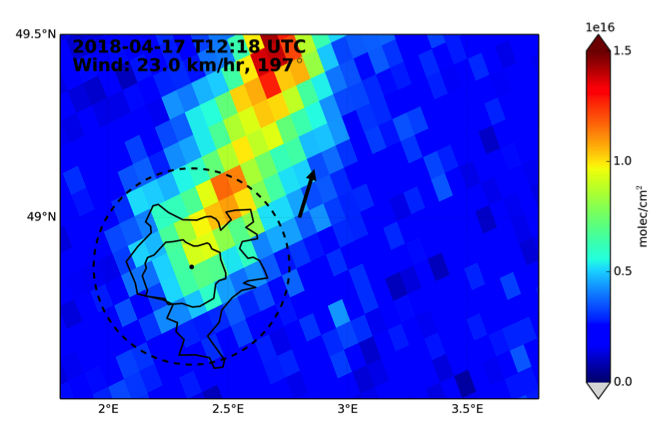 | 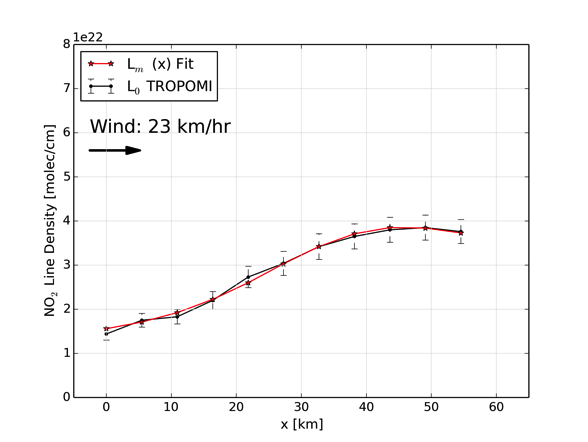 |
| 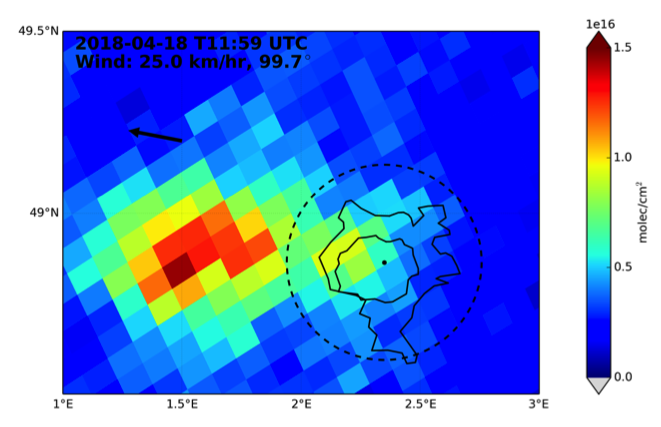 | 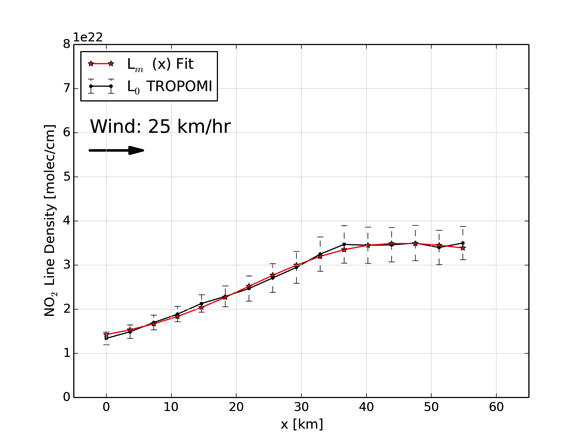 |
| 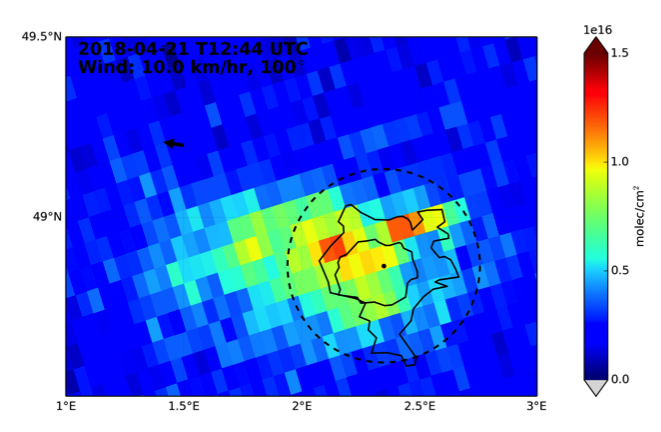 | 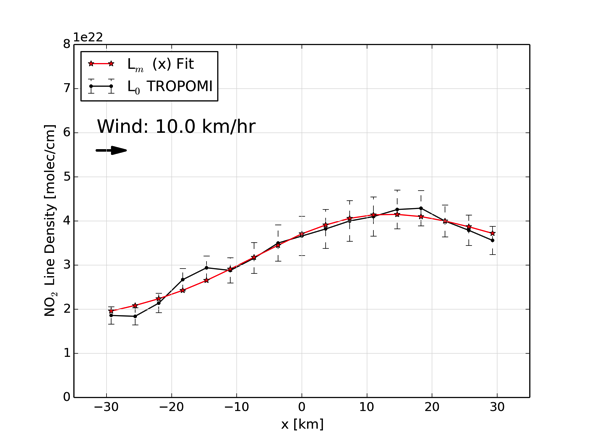 |
| 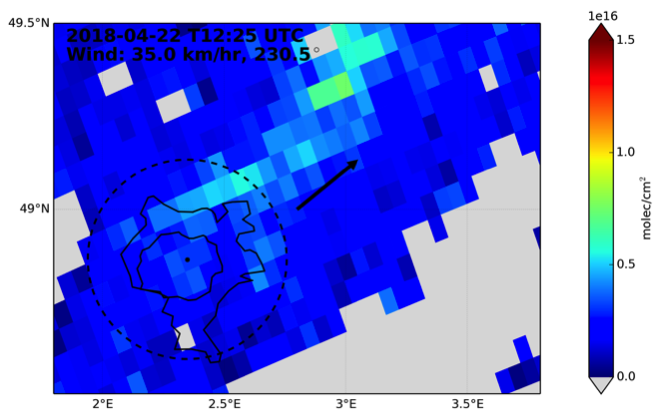 | 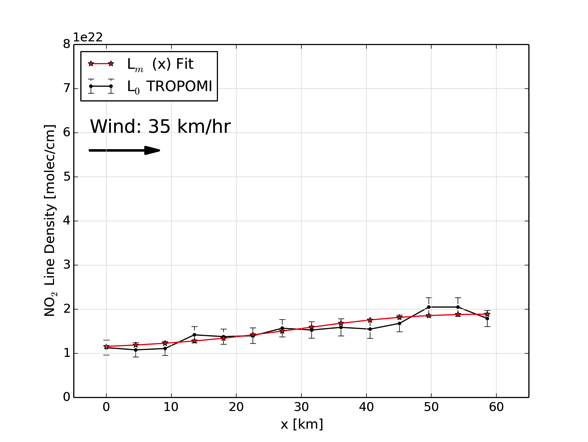 |
| 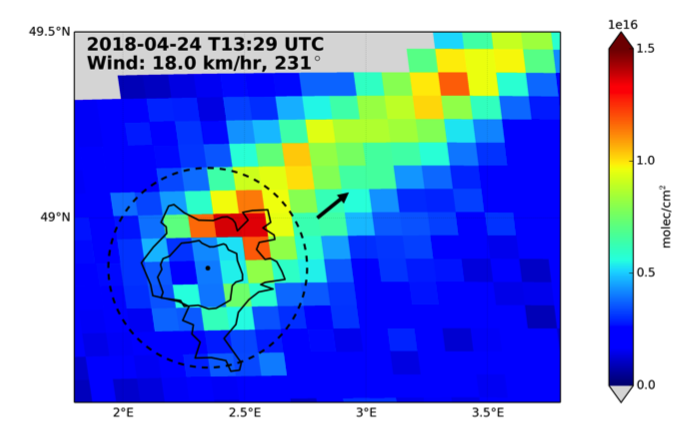 | 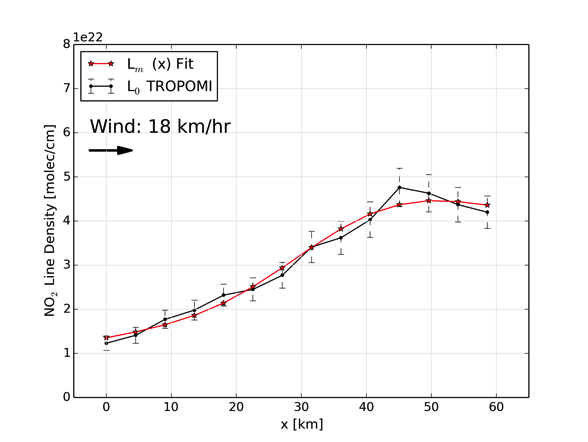 |
| **May 2018** | |
| 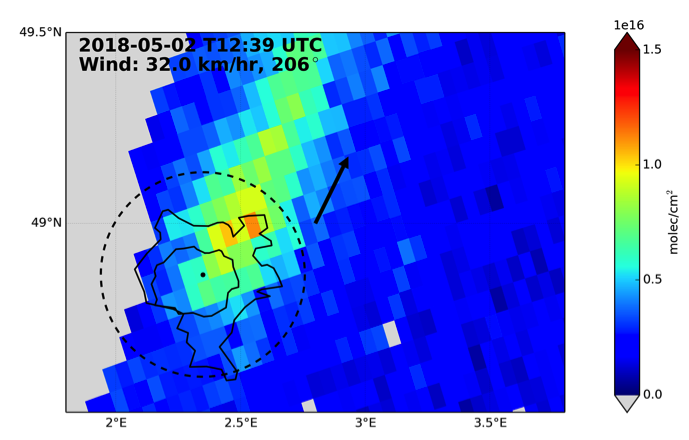 | 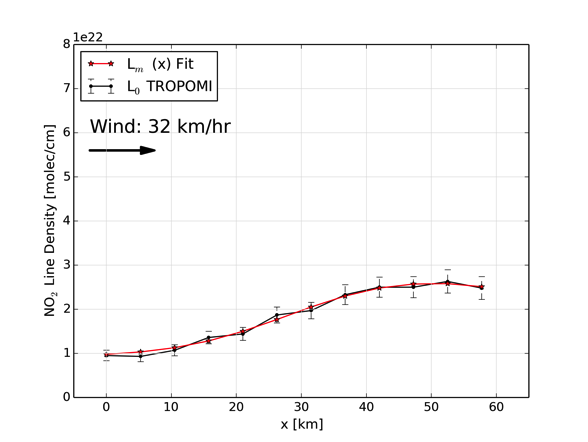 |
| 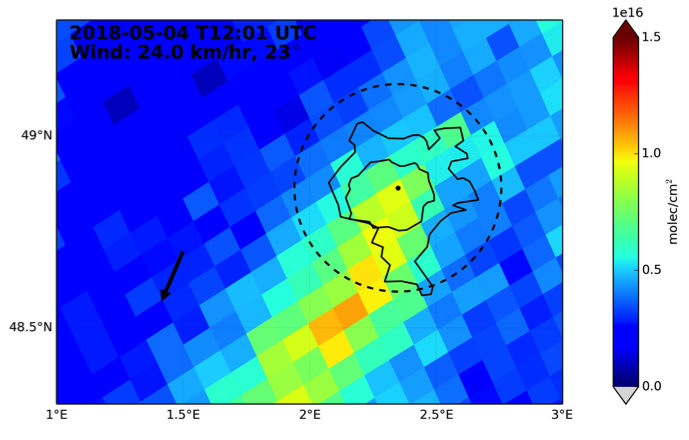 | 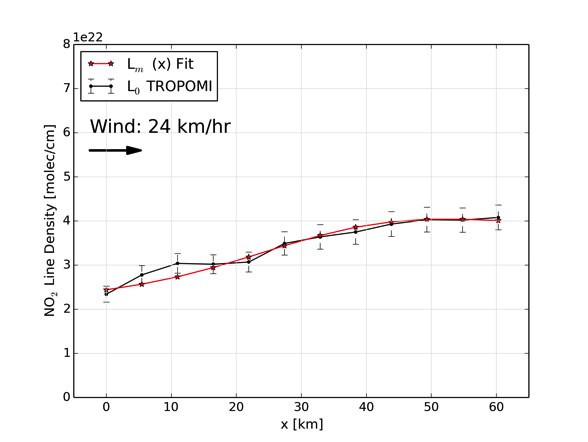 |
| 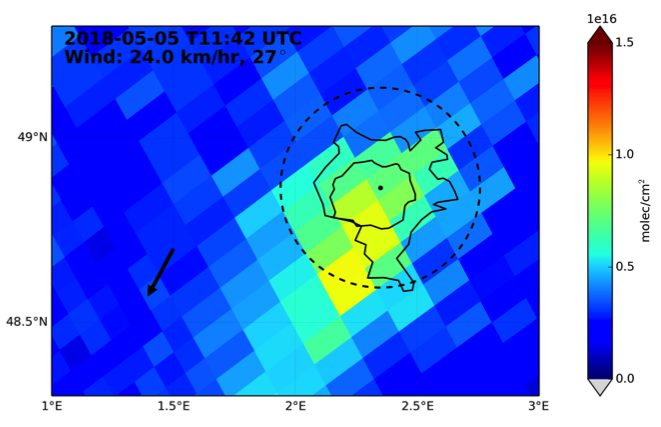 | 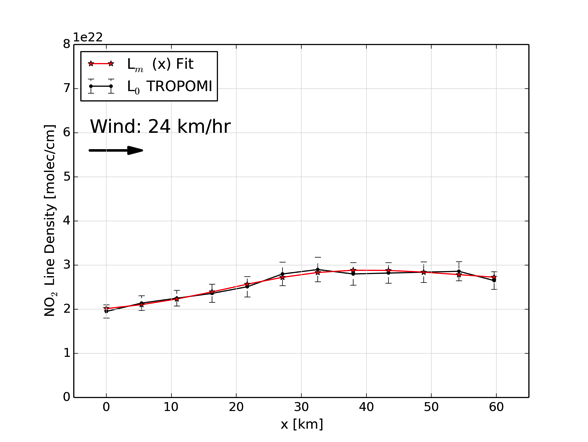 |
| 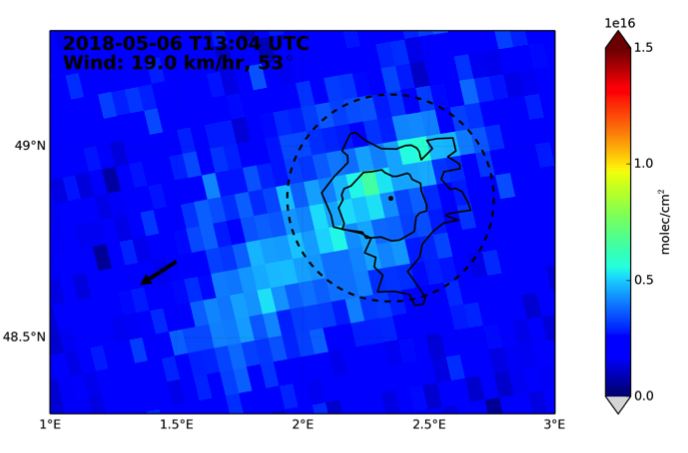 | 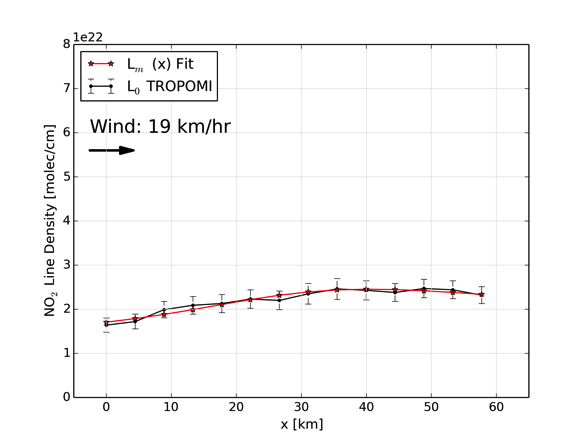 |
| 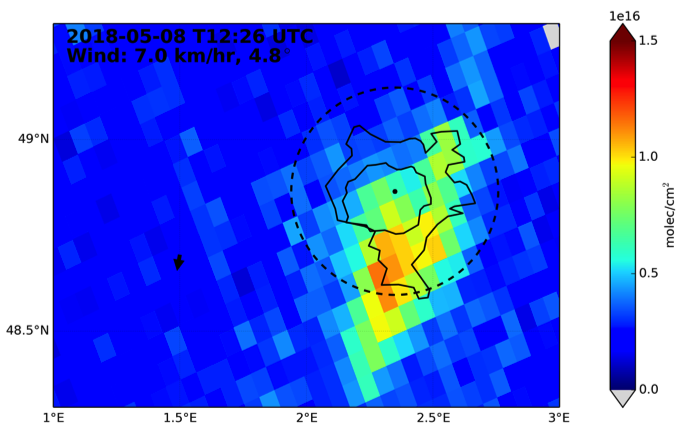 | 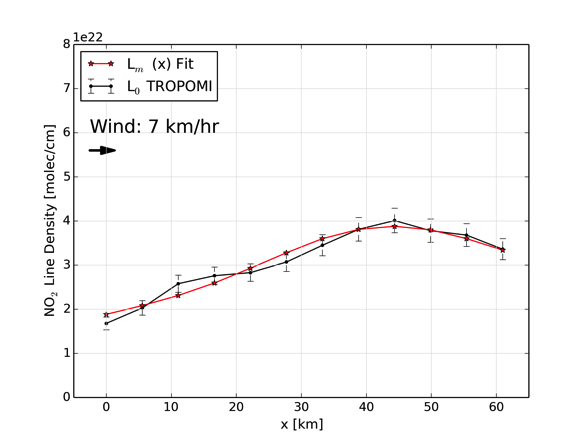 |
| 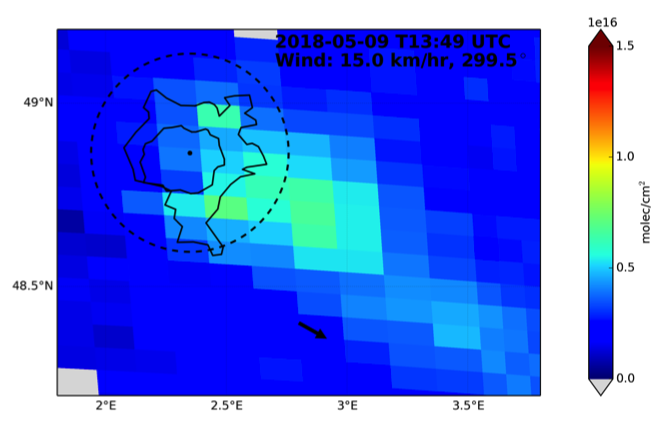 | 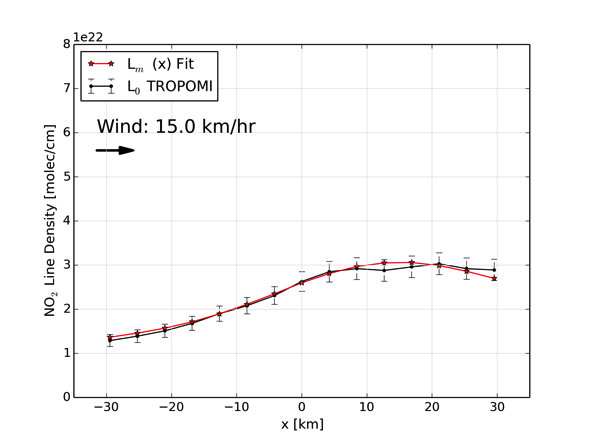 |
| 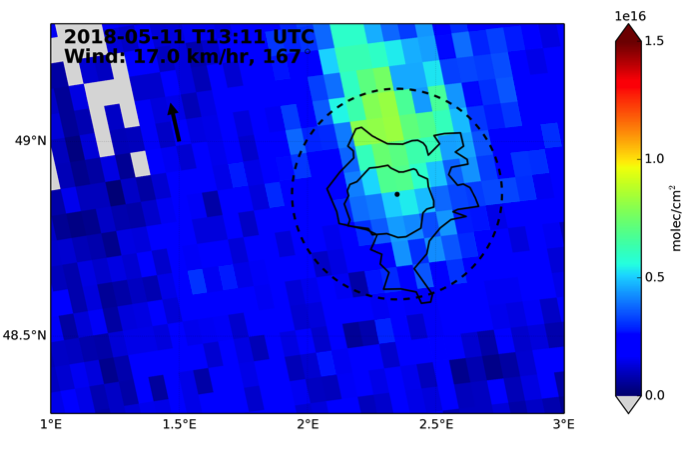 | 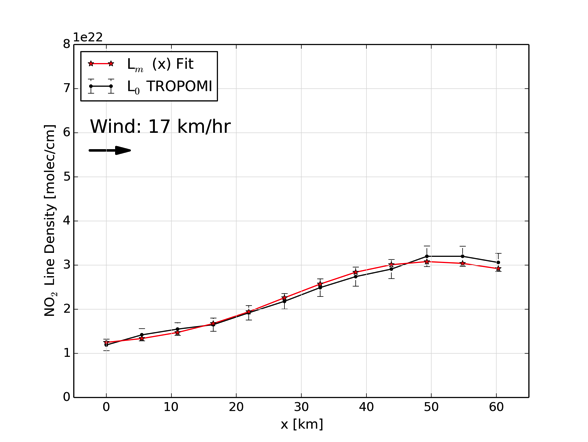 |
| 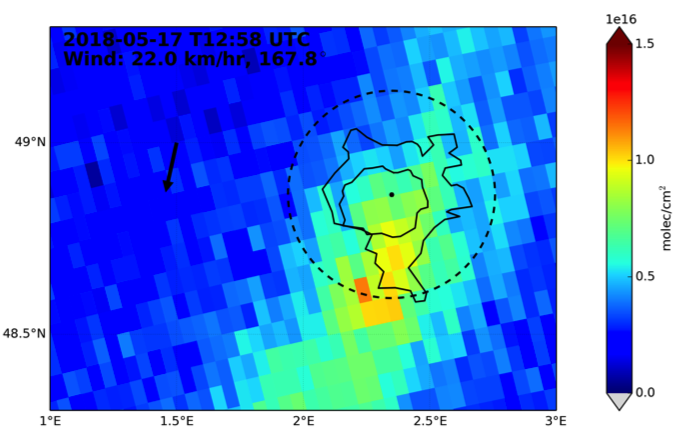 | 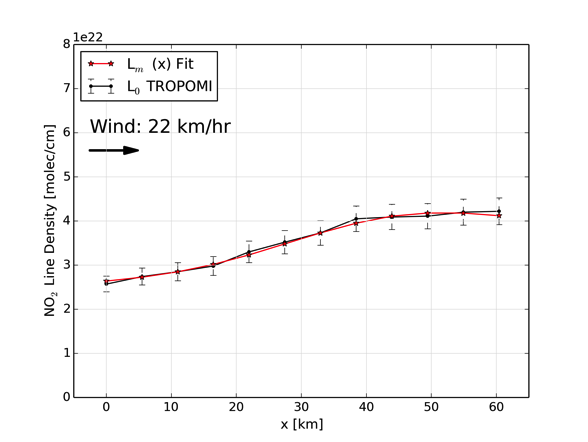 |
| 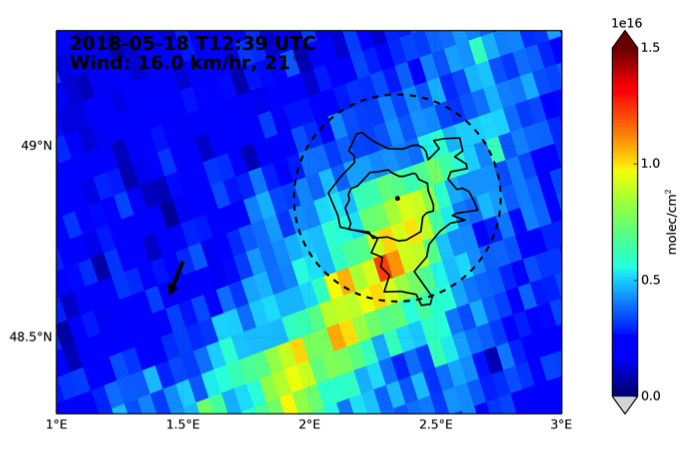 | 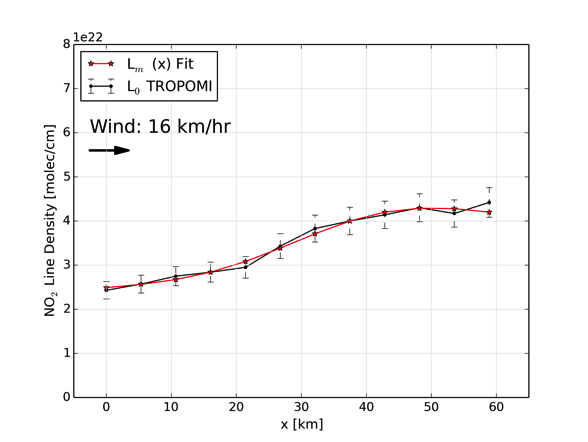 |
| 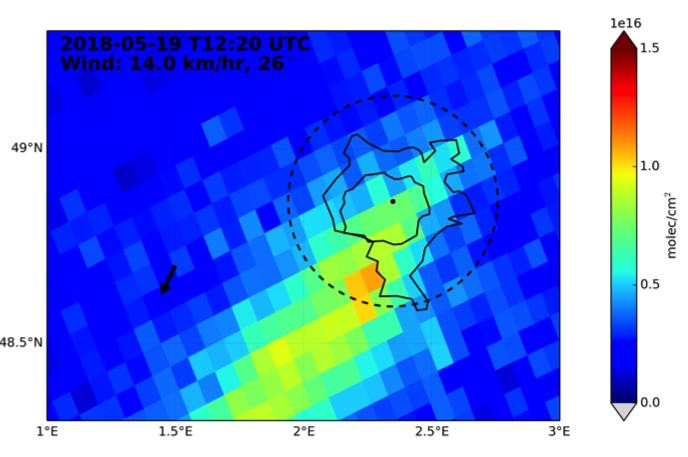 | 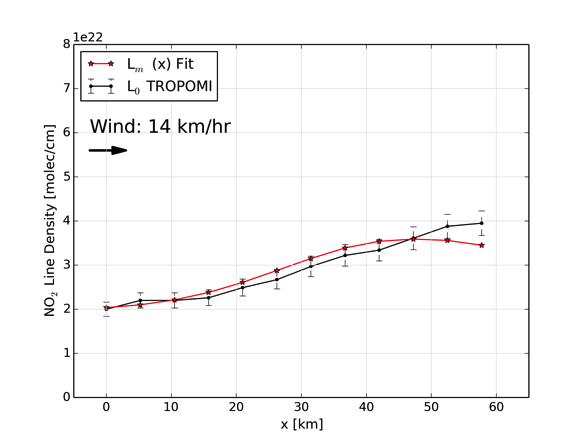 |
| **June 2018** | |
| 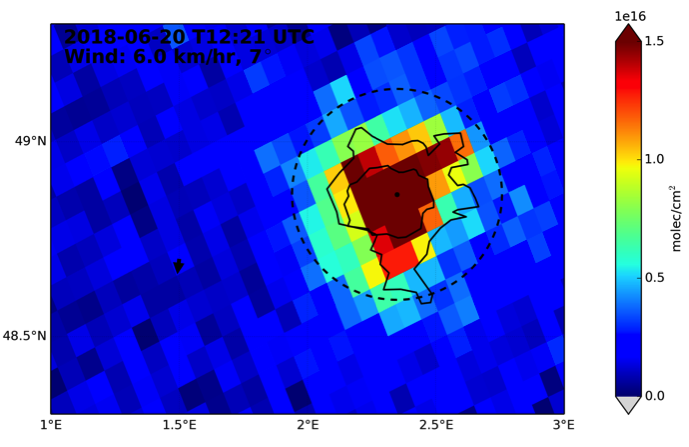 | 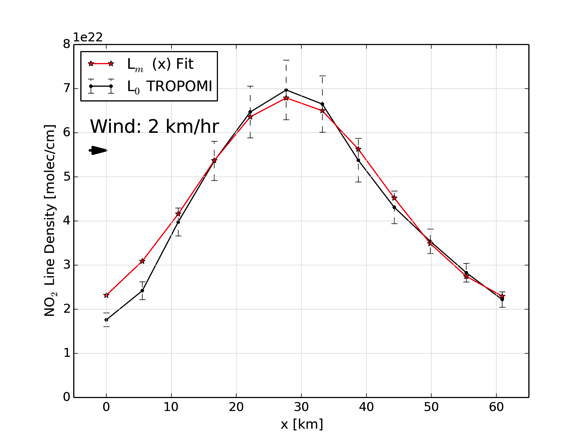 |
| 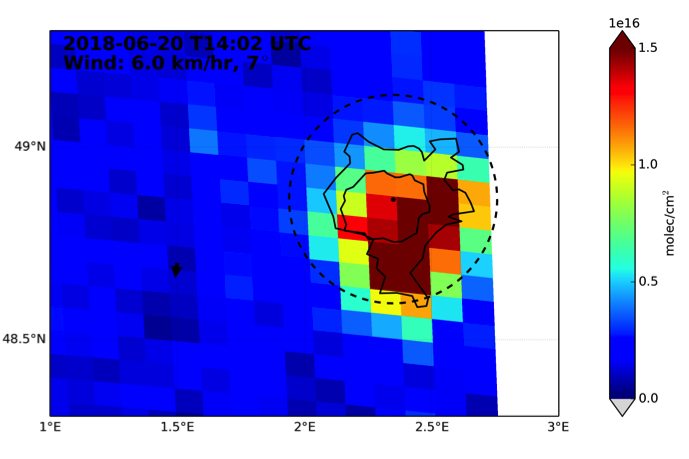 | 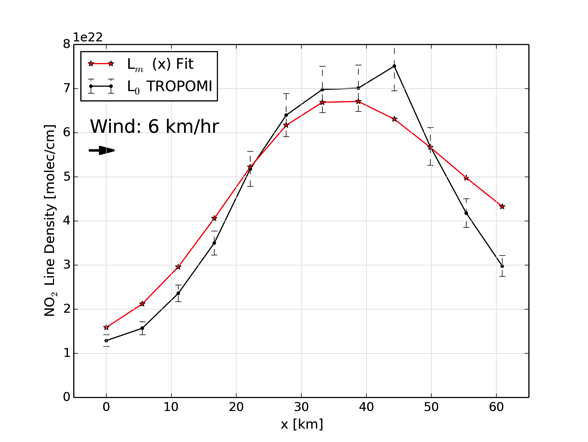 |
| 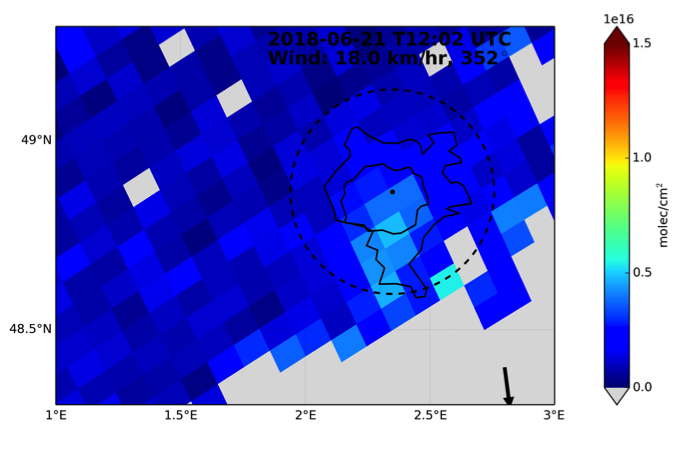 | 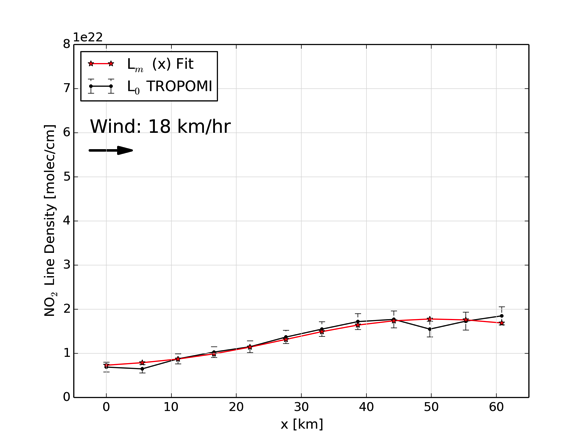 |
| 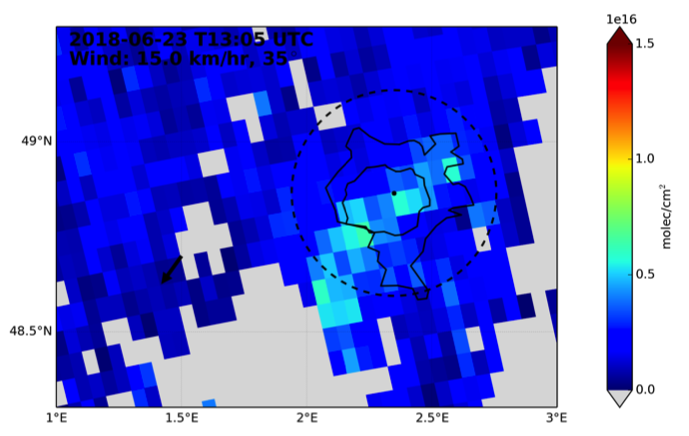 | 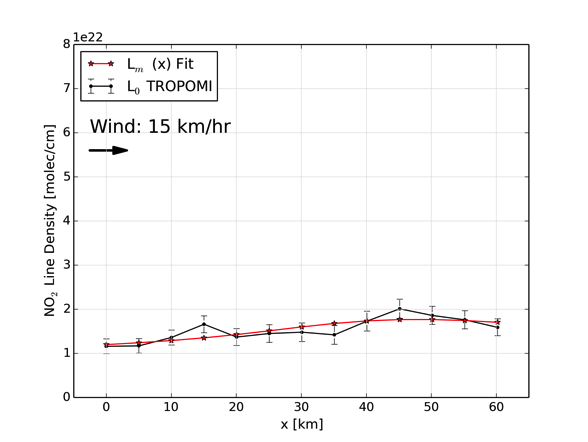 |
| 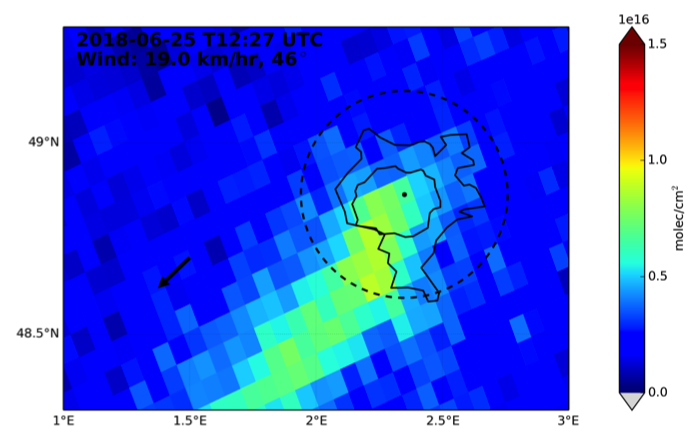 | 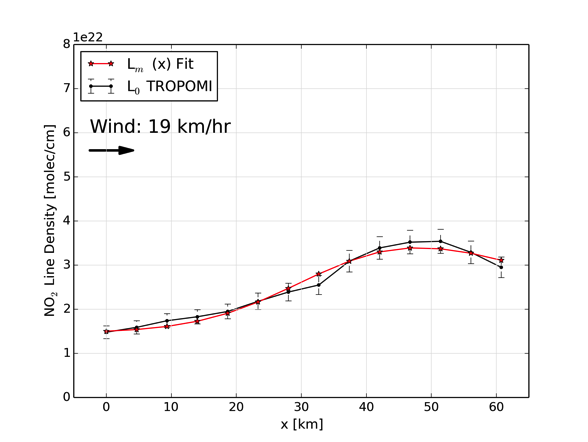 |
| 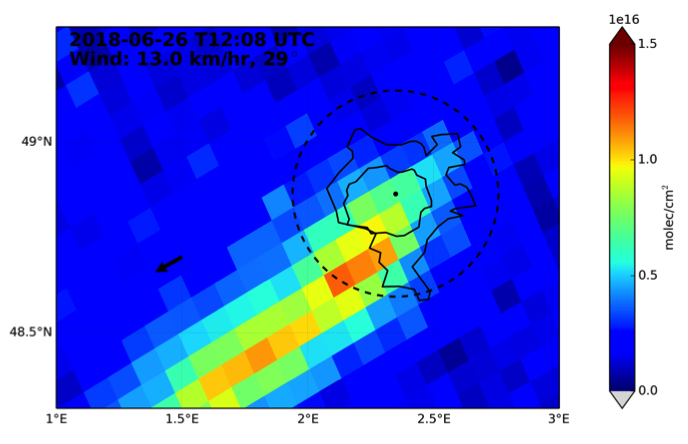 | 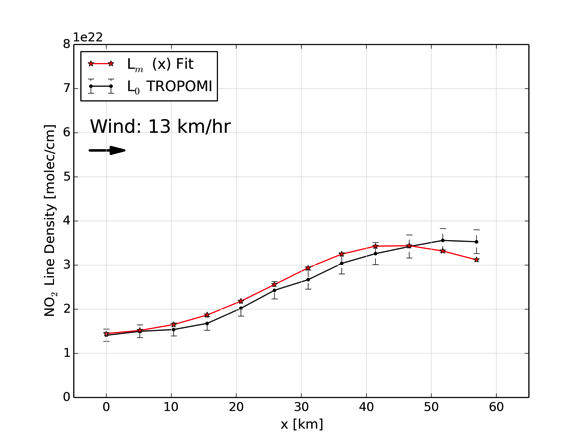 |
| 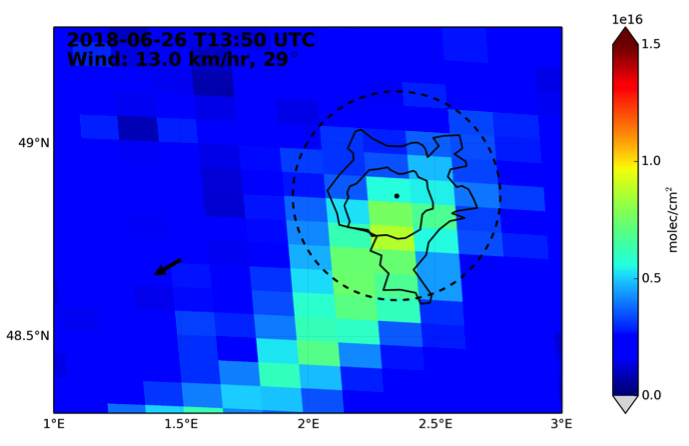 | 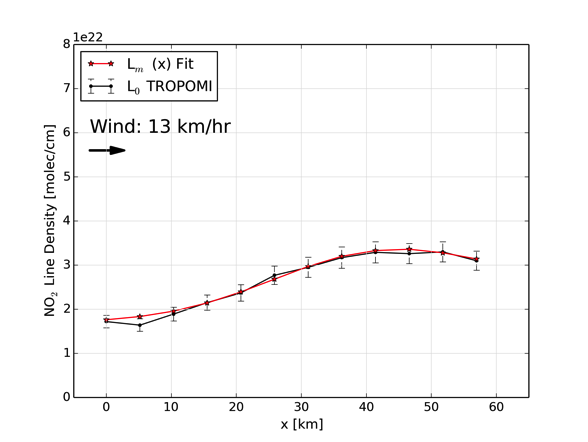 |
| 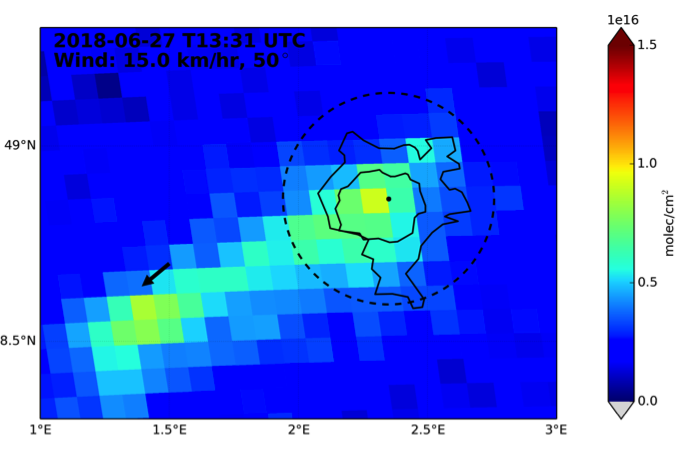 | 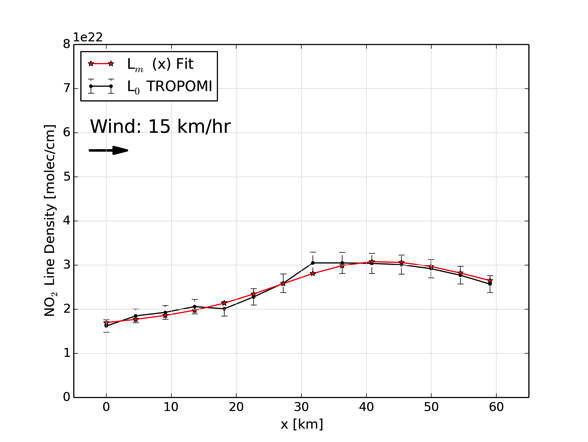 |
| 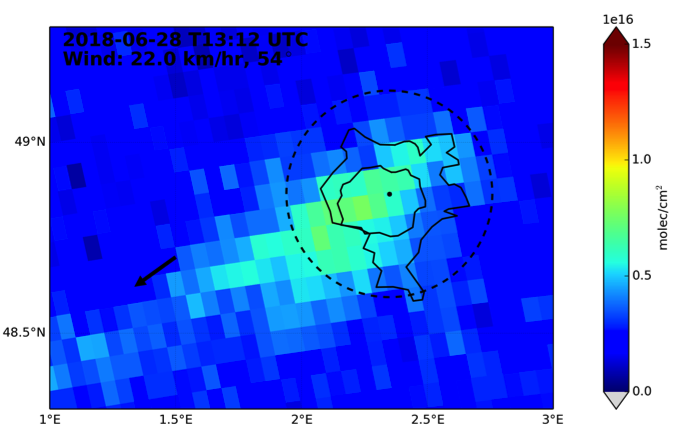 | 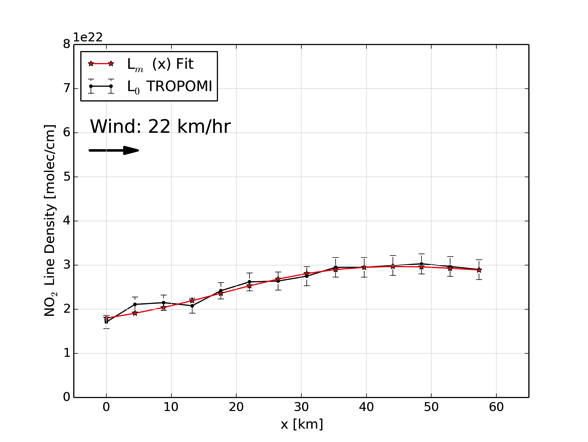 |

**Figure S6.** Tropospheric NO_2_ columns for all clear-sky days as observed by S5P-TROPOMI in the period February-June 2018 (left panels). The right panels show the corresponding observed and modeled NO_2_ line densities. The black arrow in the left panels indicates the wind direction, and the length of the arrow is proportional to the wind speed on that day at the hours just before satellite overpass.

**References**

Airparif: station data 2018, http://www.airparif.asso.fr/ (last access: 18 September 2018) (2018).

Beirle, S., Boersma, K. F., Platt, U., Lawrence, M. G., & Wagner, T.: Megacity emissions and lifetimes of nitrogen oxides probed from space. *Science*, *333*(6050), 1737-1739, 2011.

Boersma, K. F., Eskes, H. J., and Brinksma, E. J.: Error analysis for tropospheric NO_2_ retrieval from space. *Journal of Geophysical Research: Atmospheres*, *109*(D4), 2004.

Compernolle, S., Verhoelst, T., Pinardi, G., Granville, J., Lambert, J.-C., and Eichmann, K.-U., S5P MPC VDAF Validation Web Article: Nitrogen dioxide, S5P-MPC-VDAF-WVA-L2_NO2_20180904, September 2018.

Dieudonné, E., Ravetta, F., Pelon, J., Goutail, F., and Pommereau, J.-P.: Linking NO_2_ surface concentration and integrated content in the urban developed atmospheric boundary layer, *Geophys. Res. Lett., 40*, 1247-1251, doi:10.1002/grl.50242, 2013.

Kuenen, J. J. P., Visschedijk, A. J. H., Jozwicka, M., and Denier van der Gon, H. A. C.: TNO-MACC_II emission inventory; a multi-year (2003–2009) consistent high-resolution European emission inventory for air quality modelling, Atmos. Chem. Phys., 14, 10963-10976, https://doi.org/10.5194/acp-14-10963-2014, 2014.

Lorente, A., Folkert Boersma, K., Yu, H., Dörner, S., Hilboll, A., Richter, A., Liu, M., Lamsal, L. N., Barkley, M., De Smedt, I., Van Roozendael, M., Wang, Y., Wagner, T., Beirle, S., Lin, J.-T., Krotkov, N., Stammes, P., Wang, P., Eskes, H. J., and Krol, M.: Structural uncertainty in air mass factor calculation for NO_2_ and HCHO satellite retrievals, Atmos. Meas. Tech., 10, 759-782, https://doi.org/10.5194/amt-10-759-2017, 2017.

Petetin, H., Beekmann, M., Colomb, A., Denier van der Gon, H. C., Dupont, J.-C., Honoré, C., Michoud, V., Morille, Y., Perrussel, O., Schwarzenboeck, A., Sciare, J., Wiedensohler, A., and Zhang, Q. J.: Evaluating BC and NO_x_ emission inventories for the Paris region from MEGAPOLI aircraft measurements, *Atmos. Chem. Phys.*, 15, 9799-9818, doi:10.5194/acp-15-9799-2015, 2015.

Ren, X., Harder, H., Martinez, M., Lesher, R. L., Oliger, A., Simpas, J. B., ... & Zhou, X.: OH and HO_2_ chemistry in the urban atmosphere of New York City, *Atmospheric Environment*, *37*(26), 3639-3651, 2003.

Valin, L. C., Russell, A. R., and Cohen, R. C.: Variations of OH radical in an urban plume inferred from NO_2_ column measurements, Geophys. Res. Lett., 40, 1856-1860, doi:10.1002/grl.50267, 2013.

Vilà-Guerau de Arellano, J., C. C. van Heerwaarden, B. J. van Stratum, and K. van den Dries, 2015: *Atmospheric Boundary Layer: Integrating Air Chemistry and Land Interactions.* Cambridge University Press, 276 pp., doi:[https://doi.org/10.1017/CBO9781316117422](https://doi.org/10.1017%2FCBO9781316117422), 2015.

Visser, A. J., Boersma, K. F., Ganzeveld, L. N., and Krol, M. C.: European NO*_x_* emissions in WRF-Chem derived from OMI: impacts on summertime surface ozone, *Atmos. Chem. Phys., 19*, 11821–11841, https://doi.org/10.5194/acp-19-11821-2019, 2019.
